# Supplementary material for: Cytomimetic calcification in chemically self-regulated prototissues
Source: Nat Commun. 2025 May 3;16:4138. doi: 10.1038/s41467-025-59251-x (PMC12049547; doi:10.1038/s41467-025-59251-x)
Supplement: Supplementary file 1 — Supplementary Information [file 41467_2025_59251_MOESM1_ESM.pdf]

Supplementary Information

Cytomimetic calcification in chemically self-regulated prototissues

Rui Sun[1,2], Zhuping Yin[1], Molly M. Stevens[2,3], Mei Li[1]*, Stephen Mann[1,4]*

[1]*Centre for Protolife Research and Centre for Organized Matter Chemistry, School of Chemistry, University of Bristol, Bristol, United Kingdom*

[2]*Department of Medical Biochemistry and Biophysics, Karolinska Institute, Stockholm, Sweden*

[3]*Department of Physiology, Anatomy and Genetics, Department of Engineering Science, Kavli Institute for Nanoscience Discovery, University of Oxford, Oxford, United Kingdom*

[4]*Max Planck-Bristol Centre for Minimal Biology, School of Chemistry, University of Bristol, Bristol, United Kingdom*

**Email: mei.li@bristol.ac.uk; s.mann@bristol.ac.uk*

Supplementary Information:

1. Supplementary materials and methods

2. Supplementary Figures

3. Supplementary Tables

1. Supplementary Materials and Methods

Materials. All the reagents and solvents were purchased and used as received without further purification. Anhydrous dodecane, tetramethyl orthosilicate (TMOS), 3-(trimethoxysilyl)propyl methacrylate (TPM), ethanol, diethanolamine (DEA), Tris base, Tris HCl, sodium hydroxide (NaOH), calcium chloride ($CaCl_2$), poly(ethylene glycol) dimethacrylate (PEGDM, Mw 750, monomer units = 13), polyacrylic acid solution (PAA, 100 kDa, 35 wt.% in H_2O), 2-hydroxy-4'-(2-hydroxyethoxy)-2-methylpropiophenone (I2959), bovine serum albumin (BSA), hexamethylenediamine, N-hydroxysulfosuccinimide sodium salt (NHSS), 1-ethyl-3-carbodiimide hydrochloride (EDC), 2-propanol, fluorescein isothiocyanate isomer I (FITC), rhodamine B isothiocyanate (RITC), alkaline phosphatase (ALP) (≥10 DEA units/mg, 140 kDa, esterase (≥15 units/mg, 168 kDa, calcium glycerol phosphate (CaGP), ethyl acetate (EA), Alizarin Red S (ARS), FITC-dextran (40, 70, 150 kDa), orthocresolphthalein complexone (*o*-CPC), 2-amino-2-methylpropanol (AMP), 8-hydroxychinoline, disodium ethylenediaminetetraacetate (EDTA) and alginate methacrylate (10-30% methacrylation; low viscosity, Mw *ca.* 75 kDa) were purchased from Sigma-Aldrich. Dimethyl sulfoxide (DMSO), DyLight™ 405 NHS-Ester (Dy405) and DyLight™ 650 NHS-Ester (Dy650) were purchased from Thermo Fisher Scientific Inc. Sodium alginate (low viscosity, Mw *ca.* 80-120 kDa) was purchased from MP Biomedicals. Partially hydrophobic silica nanoparticles (HDK® H30) were ordered from Wacker Chemie AG. Dialysis bags with 12,000–14,000 Da were purchased from Millipore. Milli-Q-purified water (18.2 MΩ·cm) was used for all the experiments.

Dy405-labbeled sodium alginate: *Amination of Alginate (Alg-NH_2):* 120 mg sodium alginate was dissolved in 20 mL 0.1 M sodium acetic buffer (pH = 5) followed by adding 50 mg EDC and 30 mg NHSS was added into the suspension for 30 min. 60 mg hexamethylenediamine was added and reacted for 4 hours at room temperature. The mixture was precipitated in 2-propanol to remove unreacted diamine. The sample was dried at 40 °C under vacuum for overnight. *Dy405 labelling Alg-NH_2:* 10 mg Alg-NH_2 was dissolved in 5 mL 0.1 M carbonate buffer (pH = 8.5). 30 µL Dy405 in DMSO solution (2 mg/mL) was added into the suspension and reacted for overnight. The mixture was further dialyzed against with milli-Q water for at least 2 days with 3500 Da dialysis tube and freeze dried.

Dye-labelled enzymes: 10 mg/mL enzyme solution (ALP or esterase) was prepared by dissolving enzyme in 0.1 M carbonate buffer at pH = 8.5. 50 µL freshly prepared anhydrous DMSO solution of RITC or FITC (2 mg/mL) was added into enzyme solution drop wisely. The reaction mixture was stirred at 4 °C for overnight. The dye labelled enzyme solution was dialysis against milli-Q water for 2 days at 4 °C and filtered through 0.22 µm filter to remove possible aggregates and followed by freeze-drying. The acquired dye-labelled enzyme was stored at -20 °C.

ARS-staining CaP: 10 mg/mL ARS aqueous solution was firstly adjusted to pH=7.2 with ammonia/HCl and then filtered through a 0.22 µm filter. The calcified sample was washed with water for three times to remove solute. The washed sample was stained with 1 mL ARS solution for 20 min at room temperature in dark and then washed with water to remove extra ARS in the solution. The stained sample was cut into thin slice for fluorescence microscope imaging.

Colloidosome membrane permeability: A concentrated dispersion of colloidosomes was diluted 10 times (200 µL) and mixed with 200 µL of 1 mg/mL FITC-labelled dextran (or 1 mg/mL RITC- or FITC-labelled ALP). The samples were imaged with a confocal microscope after 24 hours. The interior : exterior fluorescence intensity ratio was used to evaluate the membrane permeability. The images were analysed using Image J software.

Alkaline phosphatase (ALP) activity:
Activity of free ALP and ALP after encapsulation within colloidosomes: 10 µL ALP solution (5 U/mL) together with a varied amount of p-nitrophenylphosphate (pNPP, 1 mM/5 mM) solution in buffer were

added with additional buffer solution to make the final volume up to 1 mL (ALP 0.05 U/mL). The final pNPP concentration was in the range from 2 to 4000 µM. Enzyme activity was measured using a Cary 300 UV-Vis spectrophotometer to record the change in absorbance at 410 nm. 0.2 M DEA buffer (pH 9.8), 0.2 M Tris buffer (pH 8.5 and pH 7.5) and Milli-Q water were used individually as the buffer/solvent. Activity of ALP encapsulated in colloidosomes was tested using the method described above. 10 µL ALP containing colloidosome suspension (5 U/mL) together with varied amount of pNPP (1 mM/5 mM) solution in buffer were added with addition of 0.2 M DEA buffer solution pH 9.8 to make up the final volume to 1 mL (ALP 0.05 U/mL). To avoid scattering from the colloidosome suspension, the solution was allowed to equilibrate for 2 min before recording the change in absorbance at 410 nm. **Activity of ALP in colloidosomes assembled within prototissues:** 20 µL of a tissue disk was placed in 8 mL 1 mM pNPP solution in 0.2 M DEA buffer pH 9.8 (ALP 0.05 U/mL). The sample was gently rotated on a rotator. At certain times (0/3/7/10/15/20/30/50/70 min), 100 µL solution was removed from the reaction system after centrifugation at 1249 x *g* for 30 s. The collected solution was added with additional 400 µL 0.2 M DEA buffer pH 9.8 to make up to 500 µL solution for UV-Vis spectroscopic analysis at 410 nm. To compare the ALP activity in free colloidosomes, 20 µL of a concentrated colloidosome suspension prepared by centrifugation at 555 x *g* for 2 min was added to 8 mL 1 mM pNPP solution in 0.2 M DEA buffer pH 9.8 (ALP 0.05 U/mL) and monitored at 410 nm using a Cary 300 UV-Vis spectrophotometer.

2. Supplementary Figures

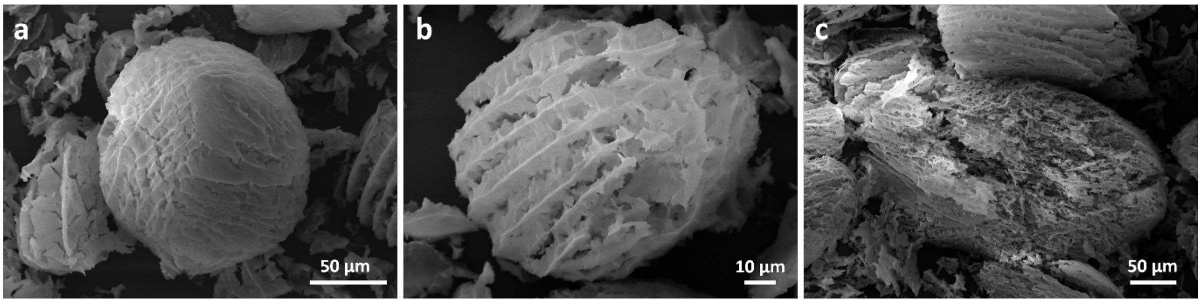

Supplementary Fig. 1. Scanning electron microscopy (SEM) images of lyophilized MA-modified colloidosomes showing silicified outer membrane (a, b) and internal silica network (c). Source data are provided as a Source Data file.

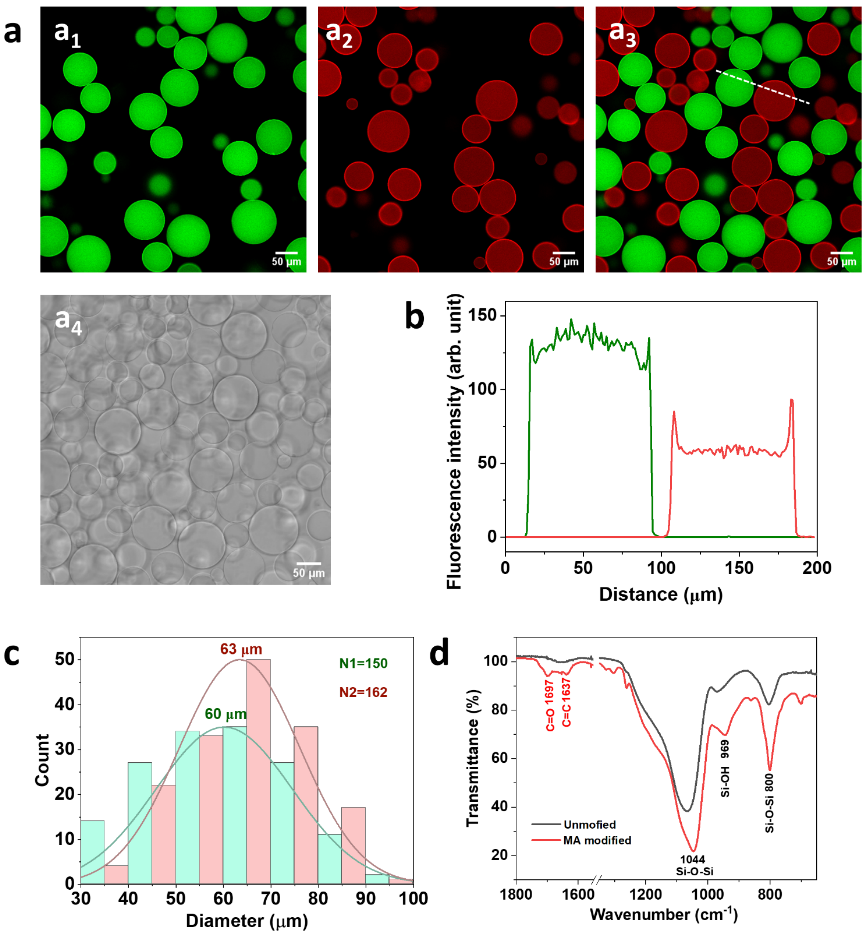

Supplementary Fig. 2. Characterization of ALP-containing MA-modified colloidosomes. (**a**) CLSM images (a_1, green channel, FITC-labelled ALP; a_2, red channel, RTIC-labeled ALP; a_3 overlay image) and bright field image (a_4). (**b**) Line profile (see dashed line in a_3) showing retention of FTIC-labeled ALP (green trace) and RTIC-labeled ALP (red trace) inside and on the membrane of the colloidosomes. (**c**) Size distribution plots for colloidosomes containing RITC-ALP (red curve) or FITC-ALP (green curve). (**d**) Fourier-transform infrared (FTIR) spectra of MA-modified colloidosomes showing characteristic MA vibration bands at 1637 cm^{-1} (C=C) and 1697 cm^{-1} (C=O), indicating successful grafting of MA groups to the colloidosome membrane. Source data are provided as a Source Data file.

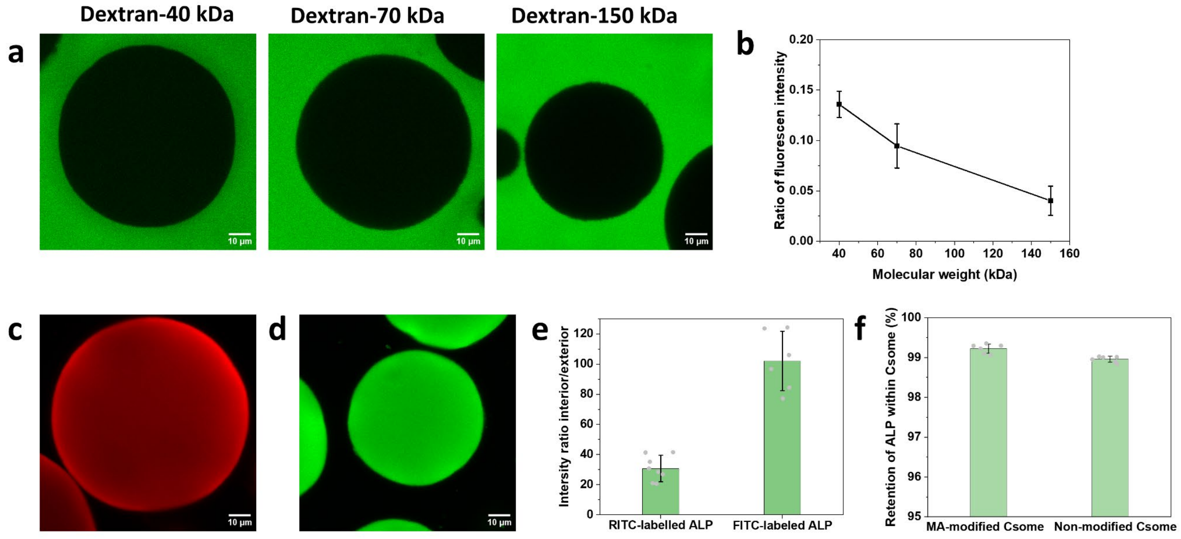

Supplementary Fig. 3. Membrane permeability of MA-modified colloidosomes. (**a**) CLSM images showing exclusion of different FITC-labeled dextrans (molecular weight (MW) = 40, 70 or 150 kDa) from the colloidosome interior after addition of the polysaccharide to the external solution. (**b**) Plot of the internal : external fluorescence intensity ratio against FITC-dextran molecular weight showing the impermeability of the MA-modified colloidosome membrane. (**c,d**) CLSM images showing uptake of RITC-ALP or FITC-ALP (M_W (ALP) = 140 kDa) from the external solution into the colloidosome interior. (**e**) Plots of internal : external fluorescence intensity ratios indicating high membrane permeability to ALP. Data are presented as mean values ± s.d. (n = 3 samples). (**f**) Plots of retention percentage of ALP determined after encapsulation within MA- or non-modified colloidosomes (Csome) showing that >99% of the total ALP is trapped inside the protocells. Data are presented as mean values ± s.d. (n = 3 samples). Source data are provided as a Source Data file.

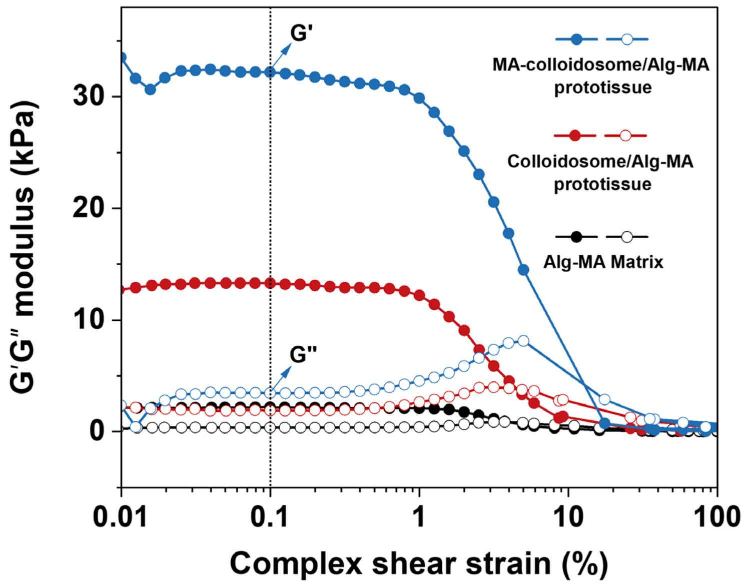

Supplementary Fig. 4. Storage modulus (G′, solid circles) and loss modulus (G′′, open circles) plots for a MA-colloidosome/Alg-MA integrated prototissue (blue line), non-modified colloidosome/Alg-MA prototissue (red line) and Alg-MA hydrogel matrix (black line). Source data are provided as a Source Data file.

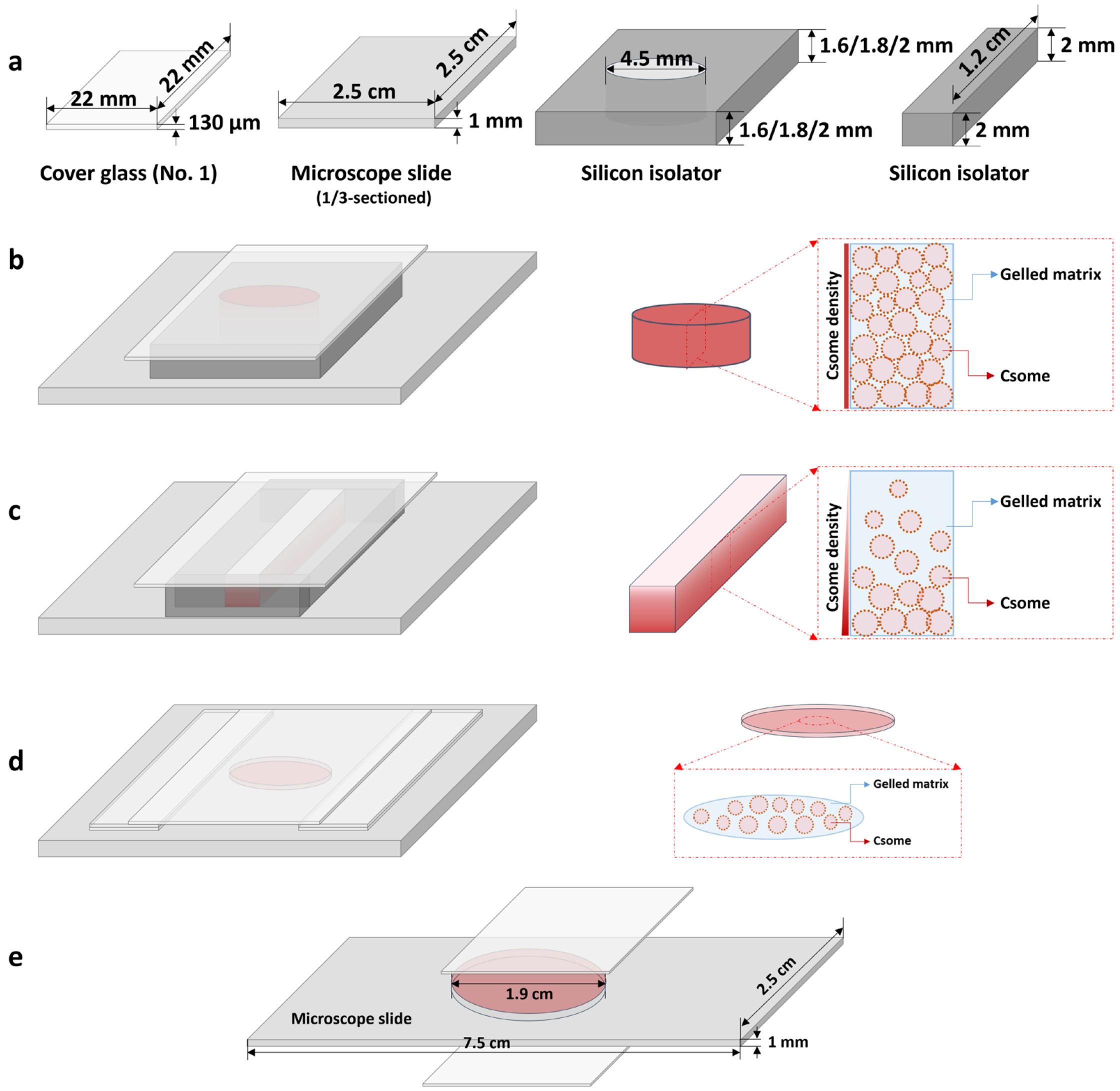

Supplementary Fig. 5. (**a-d**) Physical platforms (**a**) used for the preparation of prototissues in the form of disks (**b**), rectangular strips (**c**) and thin sheets (**d**). Parts were assembled using UV glue. Left graphics in **b-d** show illustrations of the sample holders; right graphics show corresponding prototissue arrangements. (**e**) Physical platform used for preparing samples for rheology testing. The sample was prepared in a punched microscope slide (diameter 1.9 cm x thickness 1 mm) that was covered by cover slips on both sides.

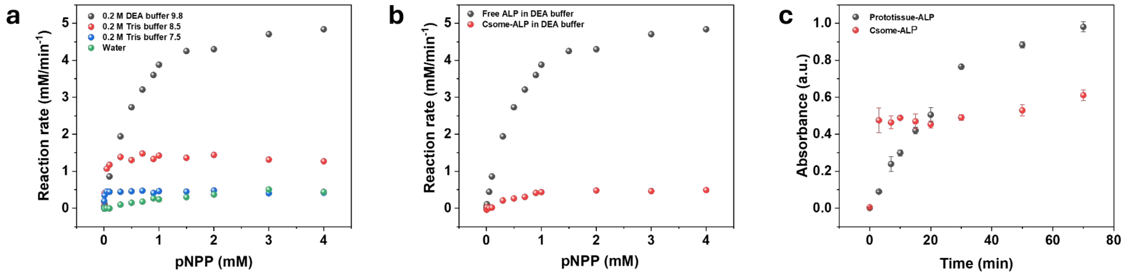

Supplementary Fig. 6. Activity of (**a**) free ALP in different aqueous buffers; (**b**) ALP after encapsulation in free colloidosomes (Csome-ALP) compared with free ALP in 0.2 M DEA buffer pH 9.8; and (c) colloidosome-encapsulated ALP within a prototissue (prototissue-ALP) compared with free Csome-ALP (n = 3 samples). The results show that ALP has the highest activity in 0.2 M DEA buffer (pH 9.8), followed by 0.2 M tris buffer (pH 8.5), 0.2 M tris buffer (pH 7.5) and Milli-Q water. ALP activity inside free colloidosomes shows a decreased activity compared with free ALP at the same conditions as in (b), possibly due to limited diffusion of the product as the colloidosomes sediment at the bottom of the quartz cuvette after 2 min. ALP activity in the prototissue shows lower activity compared with the free Csome-ALP within an initial period of 20 min (**c**). This can be attributed to the diffusion limitation placed on substrate penetration into the prototissue. After 20 min, activity in the prototissue becomes higher than in the free colloidosomes. Source data are provided as a Source Data file.

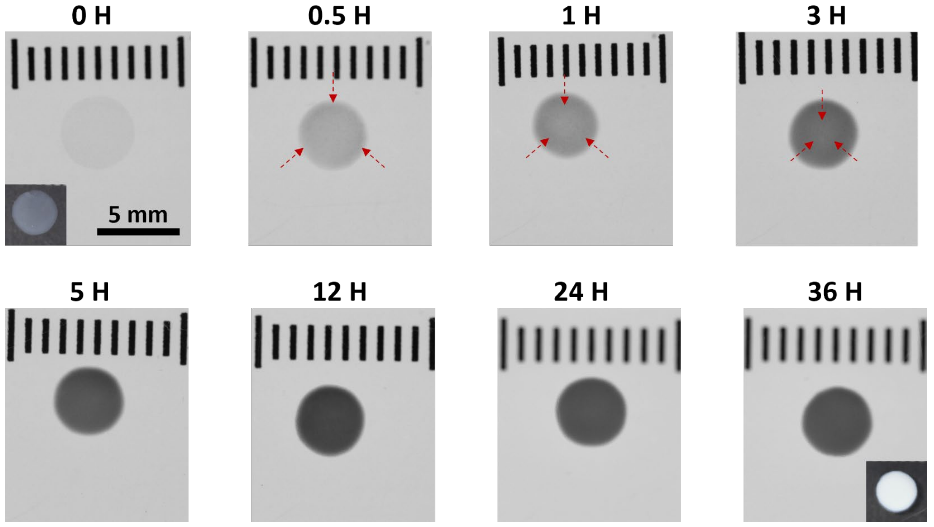

Supplementary Fig. 7. Time series of photographs of a prototissue disk undergoing endogenous calcification after addition of CaGP. Diffusion of CaGP into the translucent prototissue followed by ALP-mediated dephosphorylation inside the constituent protocells gives rise to calcium phosphate deposition. Mineralization proceeds from the disk surface inwards (indicated by red dashed arrows) to produce an opaque calcified prototissue after 24 hours. Negligible levels of shrinking (*ca*. 5 % decrease in diameter) were observed after calcification. Scale bar, 5 mm. Source data are provided as a Source Data file.

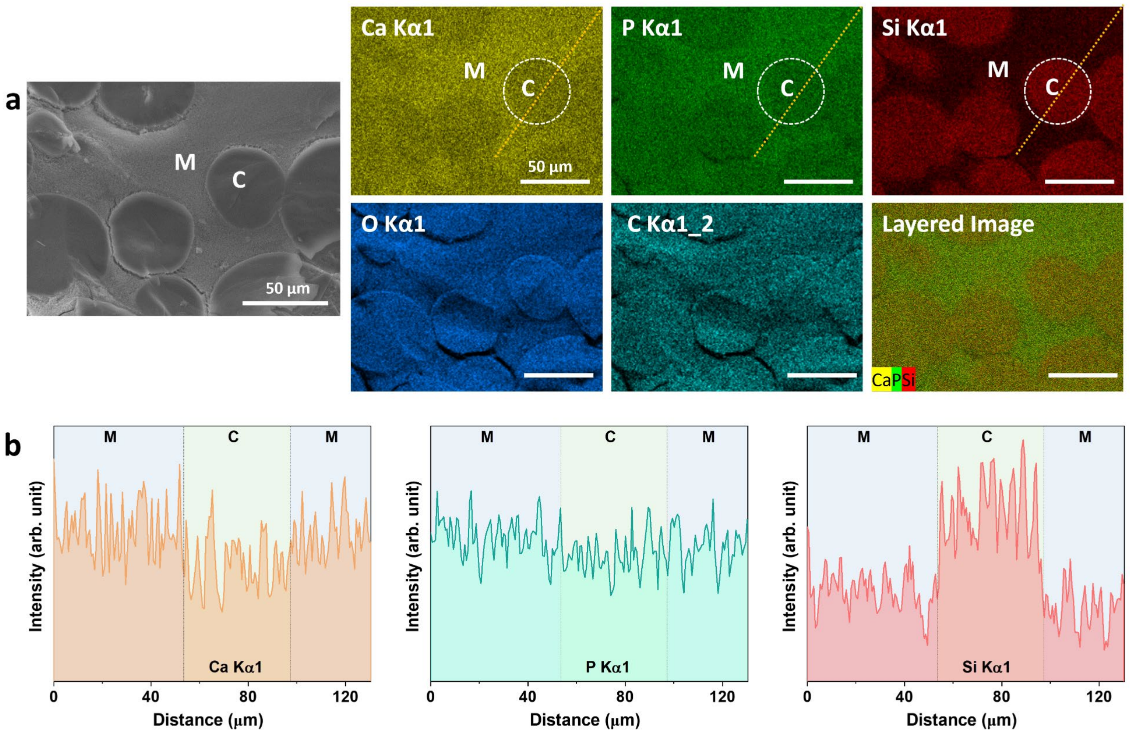

Supplementary Fig. 8. (**a**) SEM/energy dispersive X-ray (SEM-EDX) elemental mapping of a 24 h calcified prototissue disk showing Si distribution only in the MA-colloidosomes interior and Ca/P in both the MA-colloidosome interior and extra-protocellular matrix. (**b**) Corresponding Ca, P, Si line profiles (yellow dashed line in a) showing comparable distributions of Ca and P in the Alg-MA matrix (M) and MA-colloidosome (C) while Si is located specifically within C. The white dashed line indicates MA-colloidosome membrane. Source data are provided as a Source Data file.

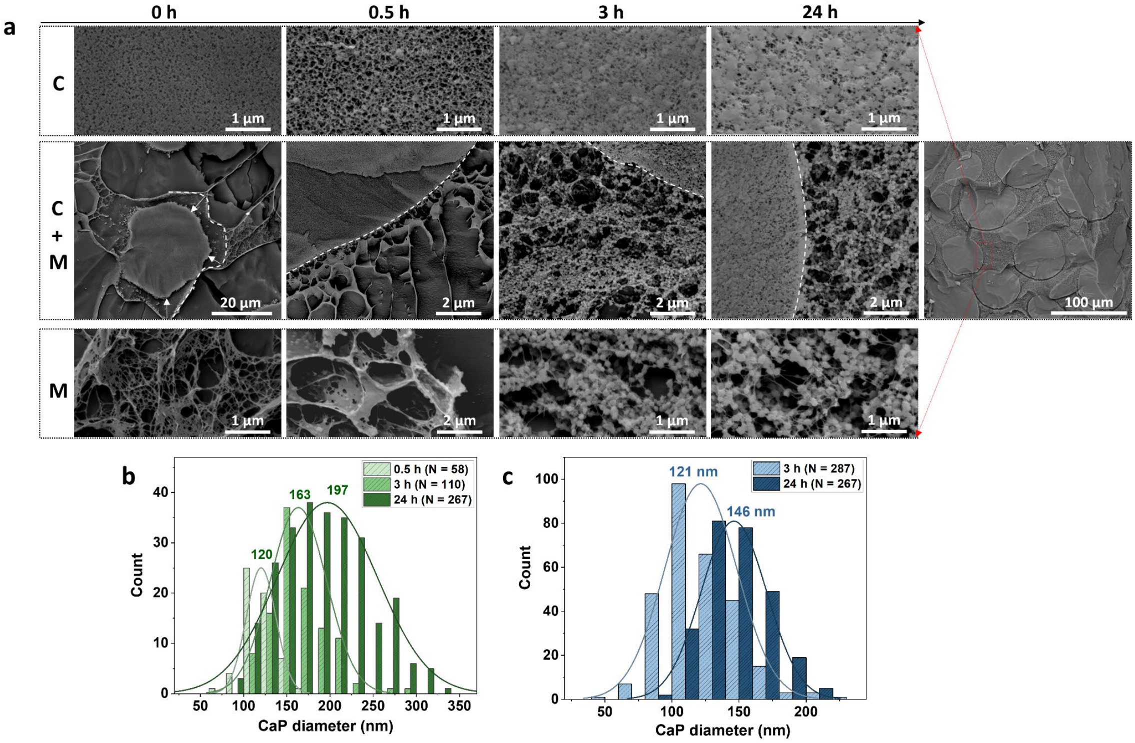

Supplementary Fig. 9. Cryo-SEM monitoring of prototissue calcification. (**a**) Images recorded from the colloidosome interior (C), extra-protocellular Alg-MA matrix (M) and at the C/M interface 0, 0.5, 3 and 24 h after addition of CaGP. Calcium phosphate particles are mainly observed in C after 0.5 h and appear initially in the matrix adjacent to C, and then throughout M after 3 h. Dense aggregates of calcium phosphate are observed with different textures in both C and M at 24 h. (**b,c**) Plots of time-dependent increases in calcium phosphate particle size in the C region (**b**) and M regions (**c**) of the prototissue. Source data are provided as a Source Data file.

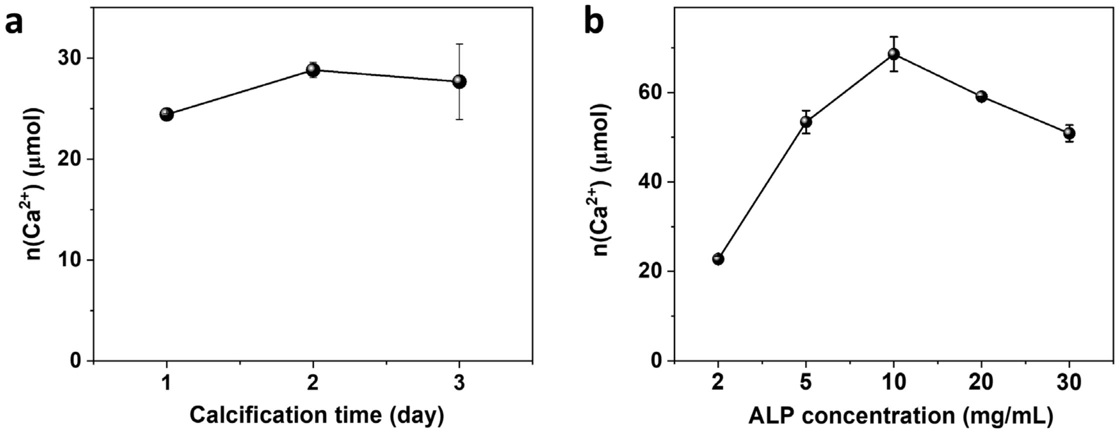

Supplementary Fig. 10. Plots of calcium content in calcified prototissues. (**a**) After calcification for 1, 2 and 3 days; and (**b**) after calcification for 1 day using different ALP concentrations within the colloidosomes. Data are presented as mean values ± s.d. (n = 3 samples). Source data are provided as a Source Data file.

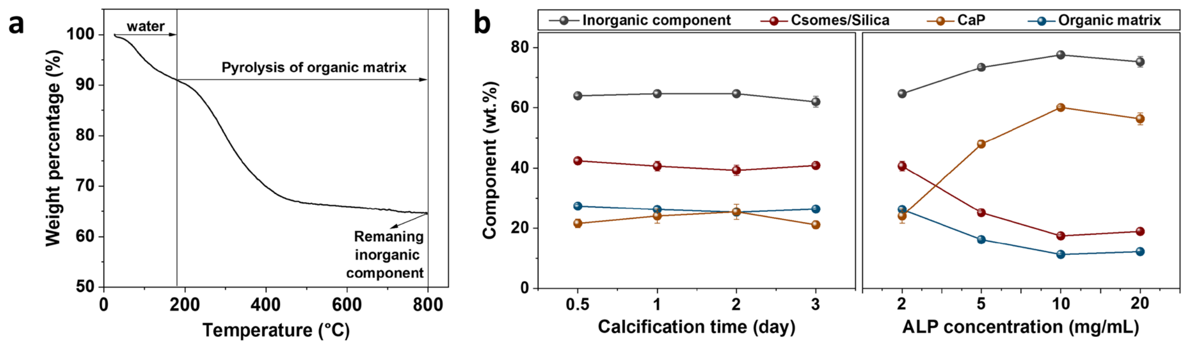

Supplementary Fig. 11. (**a**) Thermogravimetric analysis profile of a calcified prototissue. The water content was determined from the weight loss below 180 °C. The weight loss between 180 and 800 °C was ascribed to the pyrolysis of the organic matrix and the weight remaining at 800 °C corresponded to the inorganic component in the sample. In uncalcified prototissues (ref.), the remaining inorganic component was silica from the colloidosomes. The organic : silica ratio was determined as the background organic : inorganic content. For calcified prototissues, the inorganic component remaining at 800 °C consisted of both silica and calcium phosphate. The organic : silica ratio acquired from uncalcified prototissue was therefore used to determine the level of calcium phosphate in the calcified prototissues. (**b**) Plot of each component (wt.%) as a function of calcification time (left) and ALP concentration (right). Data are presented as mean values ± s.d. (n = 3 samples). Source data are provided as a Source Data file.

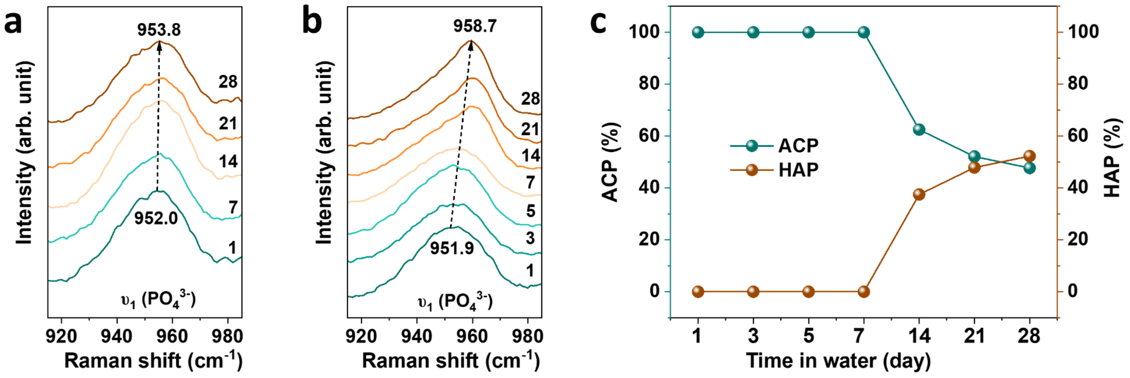

Supplementary Fig. 12. Time-dependent phase transformation of calcium phosphate in a calcified prototissue. (**a**) Time-series of Raman spectra of a calcified prototissue disk immersed in CaGP, showing retention of the amorphous calcium phosphate (ACP) phase over 28 days. A small shift in the υ_1 PO_4^{3-} vibration from 952.0 to 953.8 cm^{-1} is observed. (**b**) Time-series of Raman spectra of a calcified prototissue disk stored in pure water for 28 days showing transformation of ACP to crystalline hydroxyapatite (HAP). The υ_1 PO_4^{3-} band shifts from 951.9 cm^{-1} (ACP) to 958.7 cm^{-1} (HAP). Each spectrum is averaged from three tests on each sample. (**c**) Plot of the changes in percentage of ACP and HAP derived from Raman spectra in b by deconvolution analysis. Source data are provided as a Source Data file.

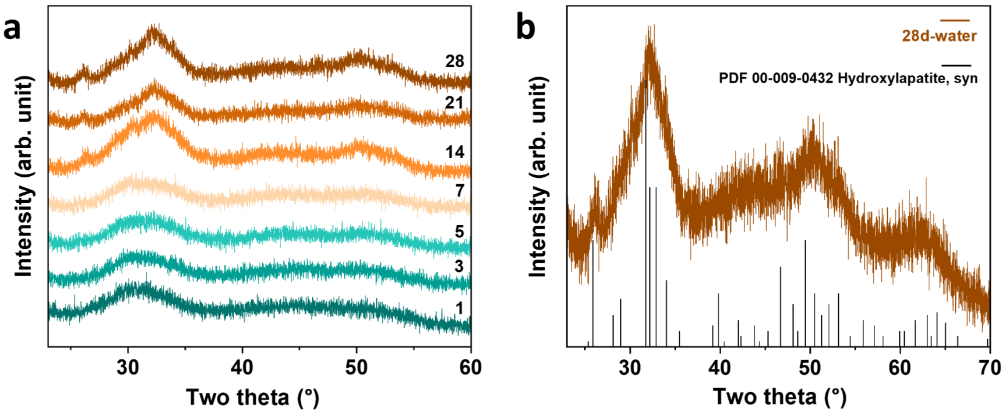

Supplementary Fig. 13. Time-dependent phase transformation of calcium phosphate in calcified prototissue disks. (**a**) Time-series of powder X-ray diffraction (PXRD) patterns of a calcified prototissue stored in water over 28 d. The initially nucleated amorphous phase gradually crystallizes into poorly crystalline HAP within 28 d. (**b**) PXRD pattern of a calcified prototissue stored in water for 28 d compared to a standard diffraction pattern of hydroxyapatite (synthesized, PDF 00-009-0432). Source data are provided as a Source Data file.

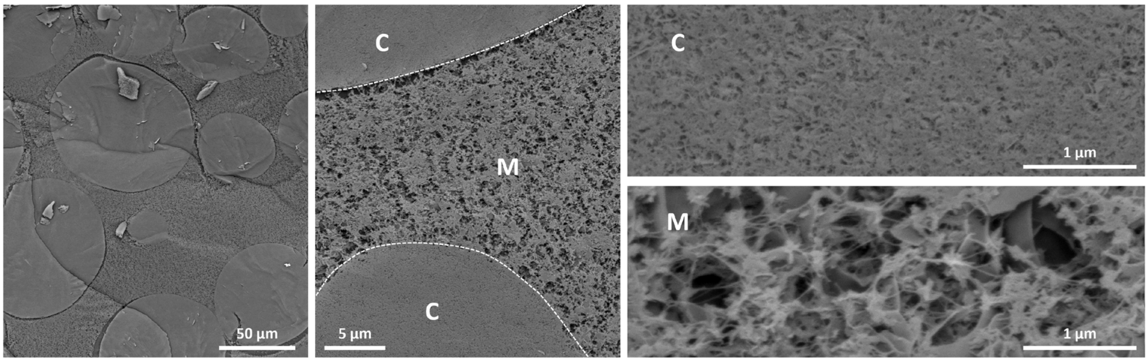

Supplementary Fig. 14. Cryo-SEM images of a 24 h calcified prototissue disk after immersion in water for 28 d showing small needle-like nanocrystals corresponding to HAP as detected by Raman and PXRD in Figures S11 and S12. C: colloidosome; M: Alg-MA matrix. White dashed line indicates the position of the colloidosome membrane. Source data are provided as a Source Data file.

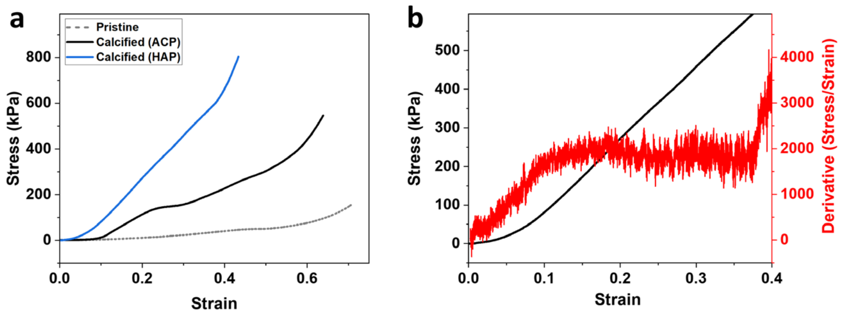

Supplementary Fig. 15. (**a**) Unconfined compression stress-strain curves of pristine (gray dashed line), calcified (ACP) (black line) and calcified (HAP) (blue line, 35 d) MA-colloidosome/Alg-MA prototissues. (**b**) Overlapping plots of stress-strain curve of calcified (ACP) prototissue (black line) and its corresponding derivative (red). Source data are provided as a Source Data file.

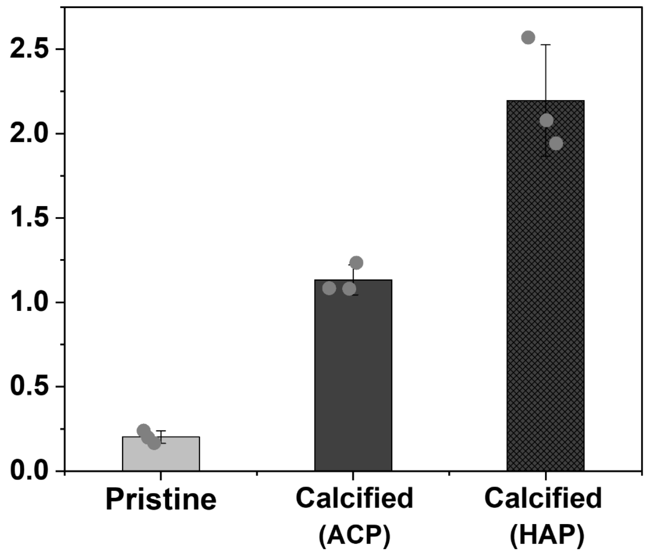

Supplementary Fig. 16. Mechanical properties of non-mineralized (pristine), calcified (ACP, 24 h) and calcified (HAP, 35 d) prototissues measured by unconfined universal compression. The encapsulated ALP concentration is 2 mg/mL. Data are presented as mean values ± s.d. (n = 3 samples). Source data are provided as a Source Data file.

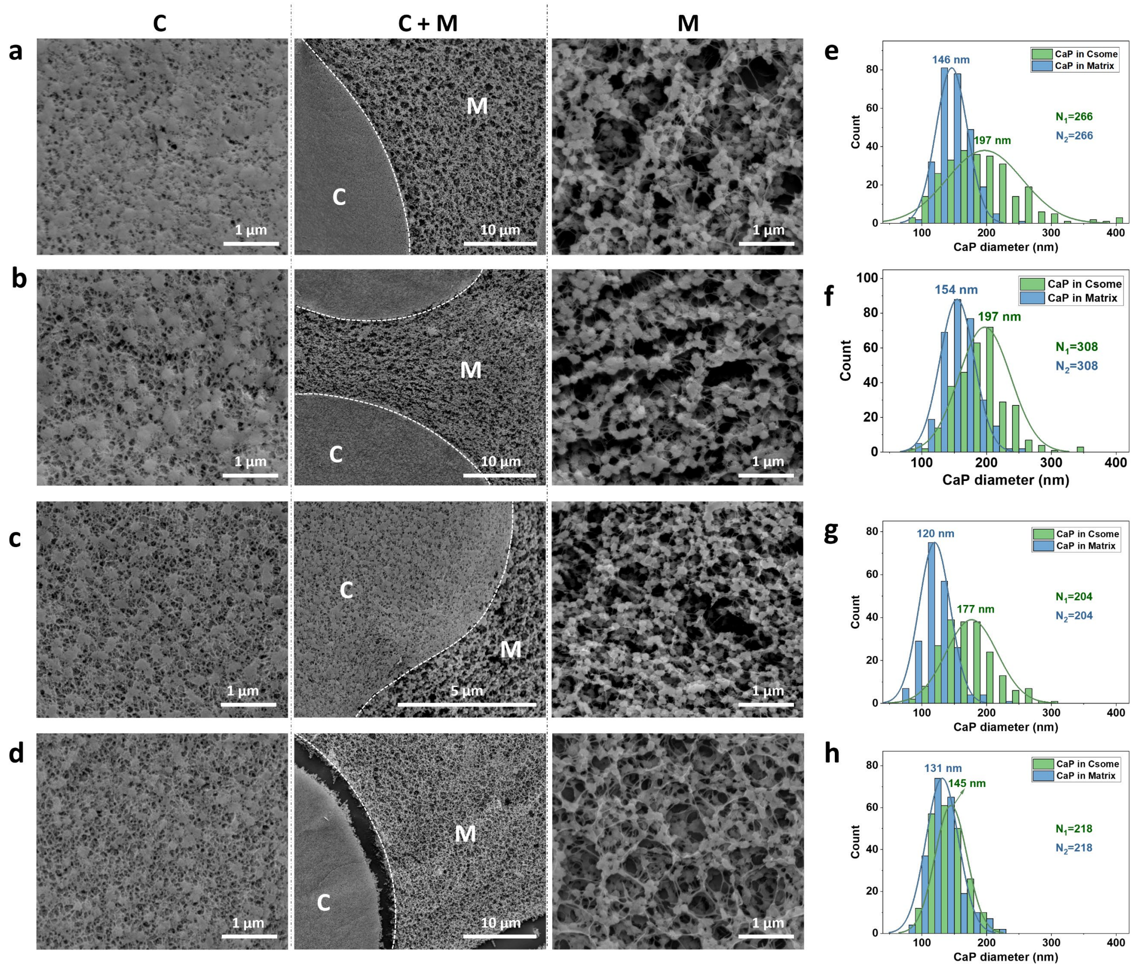

Supplementary Fig. 17. Cryo-SEM backscattered images of 24 h calcified prototissues prepared with different Alg-MA/PEGDM matrix compositions: (**a**) PEG, 0 wt.%, (**b**) PEG, 25 wt.%, (**c**) PEG, 50 wt.% and (**d**) PEG, 75 wt.%. (**e-h**) Corresponding plots of calcium phosphate (CaP) particle diameter distributions. The images show that the CaP particle diameter and number density within the colloidosomes (Csomes) gradually decrease as the PEG matrix content is increased. Specifically, calcified prototissues prepared with 50 wt.% PEGDM comprised a continuous network of calcium phosphate particles *ca*. 177 (interior) and 120 nm (matrix) in size, while discrete mineral clusters were embedded in a relatively high-density polymer matrix at 75 wt. % PEGDM. The white dashed lines indicate the position of the MA-colloidosome membrane. C: MA-colloidosome; M: Alg-MA/PEGDM matrix. Source data are provided as a Source Data file.

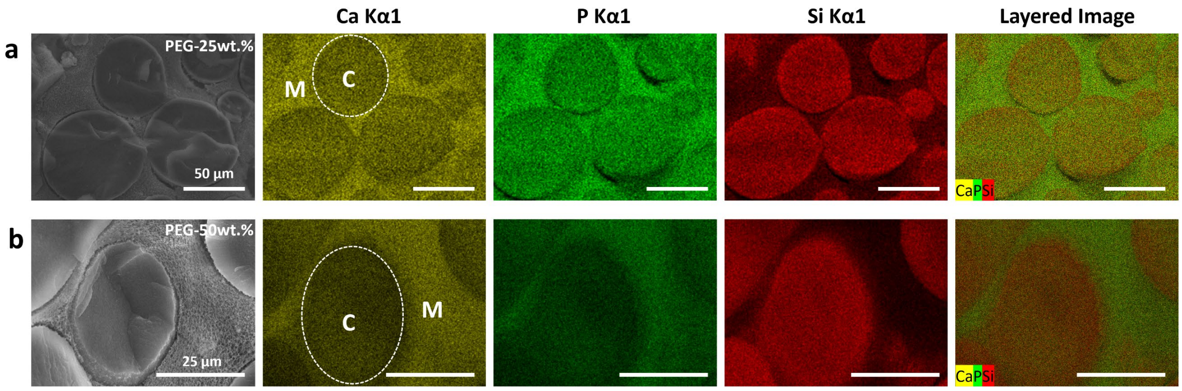

Supplementary Fig. 18. SEM-EDX elemental mapping of calcified prototissues fabricated with different levels of PEGDM in the Alg-MA/PEGDM matrix. (**a**) PEG, 25 wt.% and (**b**) PEG, 50 wt.% showing Ca and P distributions throughout the prototissue. The Ca and P intensities within the colloidosomes gradually decrease with increasing levels of PEG in the matrix. The white dashed lines indicate the position of the MA-colloidosome membrane. Source data are provided as a Source Data file.

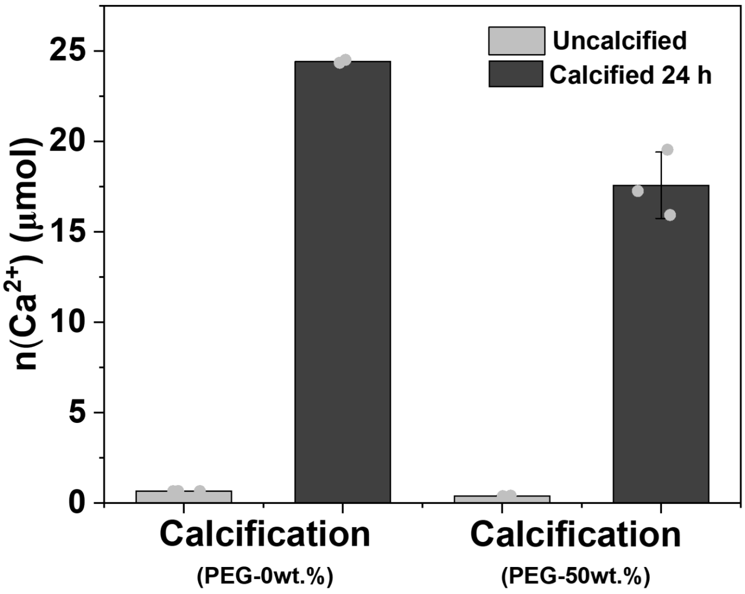

Supplementary Fig. 19. Plot showing amount of Ca^{2+} determined from acid-extracted prototissues prepared with 0 wt.% and 50 wt.% PEG in a Alg-MA/PEGDM matrix; the Ca^{2+} measurements are used as a proxy for the amount of calcium phosphate deposited in the prototissue. Ca^{2+} concentrations of 24.42 ± 0.11 and 17.57 ± 1.83 µmol were determined for calcified prototissues prepared at 0 and 50 wt.% PEGDM, respectively, suggesting that the presence of PEG inhibited calcium phosphate mineralization. Data are presented as mean values ± s.d. (n = 3 samples). Source data are provided as a Source Data file.

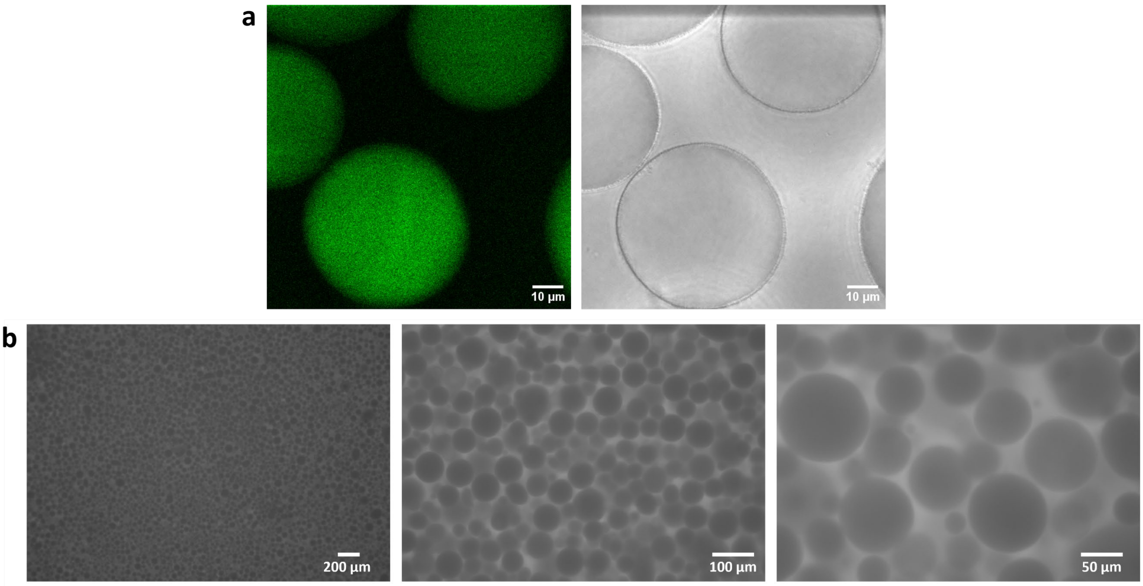

Supplementary Fig. 20. (**a**) CLSM fluorescence microscopy (left) image showing the presence of intact enzyme-containing colloidosomes (green fluorescence, FITC-labelled ALP) within a crosslinked PEGDM matrix (black regions). Corresponding BF image is shown in the right panel. Scale bars, 10 µm. (**b**) Bright field images of a mineralized prototissue recorded at different magnifications showing a high number density of calcified colloidosomes (discontinuous dark domains) embedded in a continuous mineral-free PEGDM matrix (continuous light regions). Scale bars, 200 (left), 100 (middle) and 50 µm (right).

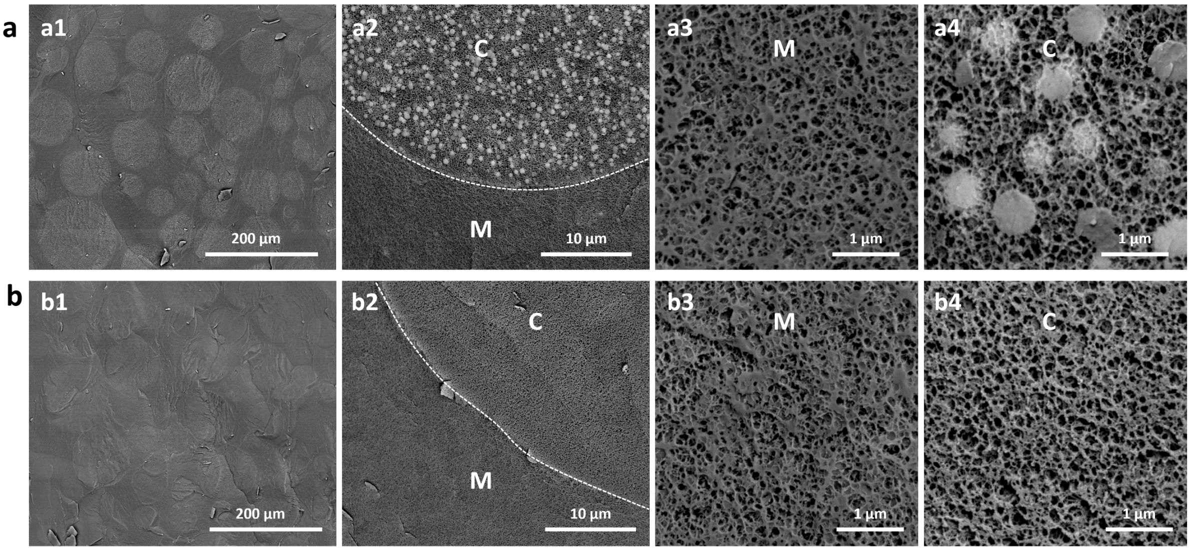

Supplementary Fig. 21. (**a**) Cryo-SEM images of a freeze-fractured 24 h calcified prototissue showing mineralized colloidosomes (light grey domains) embedded in a continuous mineral-free matrix (a1), the interface between an individual calcified colloidosome (C) and surrounding PEGDM matrix (M) (a2), and the corresponding magnified images recorded respectively in the M (a3) and C regions (a4) of the calcified protocell/matrix-integrated prototissue. (**b**) Cryo-SEM images of a non-calcified colloidosome/PEGDM integrated prototissue (b1), the interface between C and M (b2), and the corresponding magnified images recorded respectively in the M (b3) and C regions (b4) showing a dense PEGDM matrix network and silica network within the colloidosome interior. Source data are provided as a Source Data file.

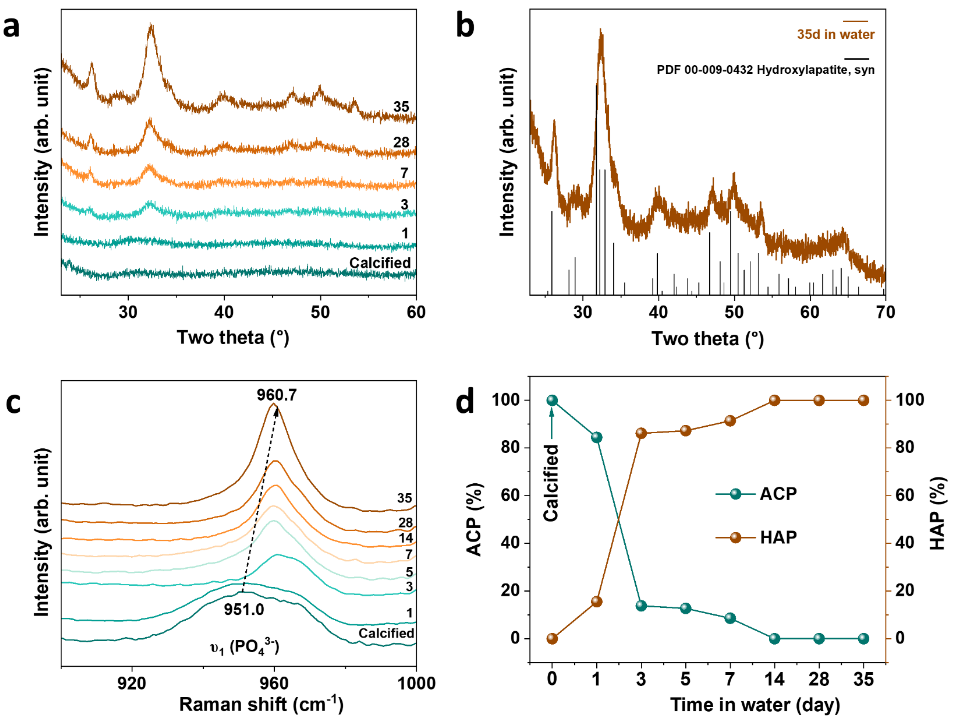

Supplementary Fig. 22. Time-dependent phase transformation of calcium phosphate in a prototissue with selective intra-protocellular mineralization. (**a**) Time-series of PXRD patterns of an intra-protocellular calcified prototissue disk stored in water over 35 d. The initially ACP phase crystallizes into poorly crystalline HAP in 3 d and the ACP to HAP transformation is complete within 35 d. (**b**) PXRD pattern of a calcified prototissue stored in water for 35 d compared to a standard diffraction pattern of hydroxyapatite (synthesized, PDF 00-009-0432). (**c**) Time-series of Raman spectra of an intra-protocellular calcified prototissue disk stored in pure water for 35 d showing transformation of ACP to crystalline HAP. The $\upsilon 1$ PO_4^{3-} band shifts from 951.0 cm^{-1} (ACP) to 960.7 cm^{-1} (HAP). Each spectrum is averaged from three tests on each sample. (**d**) Plot of the changes in percentage of ACP and HAP contents in intra-protocellular calcified prototissue derived from Raman spectra in **c** by deconvolution analysis. Source data are provided as a Source Data file.

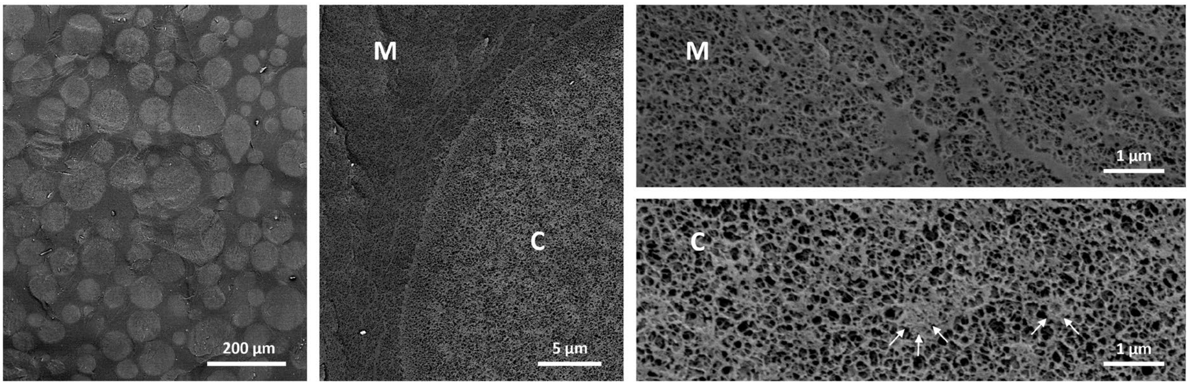

Supplementary Fig. 23. Cryo-SEM images of a freeze-fractured intra-protocellular calcified prototissue after immersion in water for 35 days. Spherical ACP particles are deposited within the colloidosomes (C) and subsequently transform into poorly crystalline HAP particles (indicated by the white arrows). C: colloidosome; M: Alg-MA matrix. Source data are provided as a Source Data file.

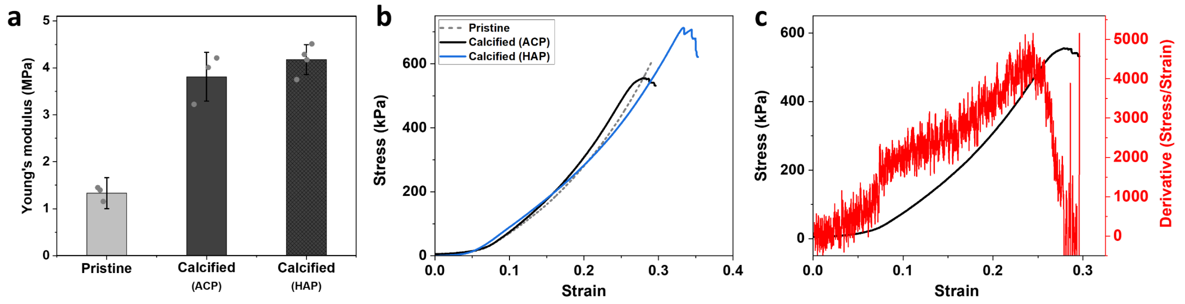

Supplementary Fig. 24. (**a**) Mechanical properties of MA-colloidosome/PEGDM-integrated prototissues with selective intra-protocell calcification; non-mineralized (pristine), calcified with ACP (24 h) and after ACP transformation to HAP (35 d) prototissues. Samples measured by unconfined universal compression. Error bars represent standard deviation (n = 3 samples for Pristine and Calcified-ACP; n = 4 samples for calcified-HAP). (**b**) Unconfined compression stress-strain curves of pristine (gray dashed line), calcified (ACP) (black line) and calcified (HAP) (blue line) MA-colloidosome/PEGDM-integrated prototissues. (**c**) Overlapping plots of stress-strain curve of calcified (ACP) prototissue (black line) and its corresponding derivative (red). Source data are provided as a Source Data file.

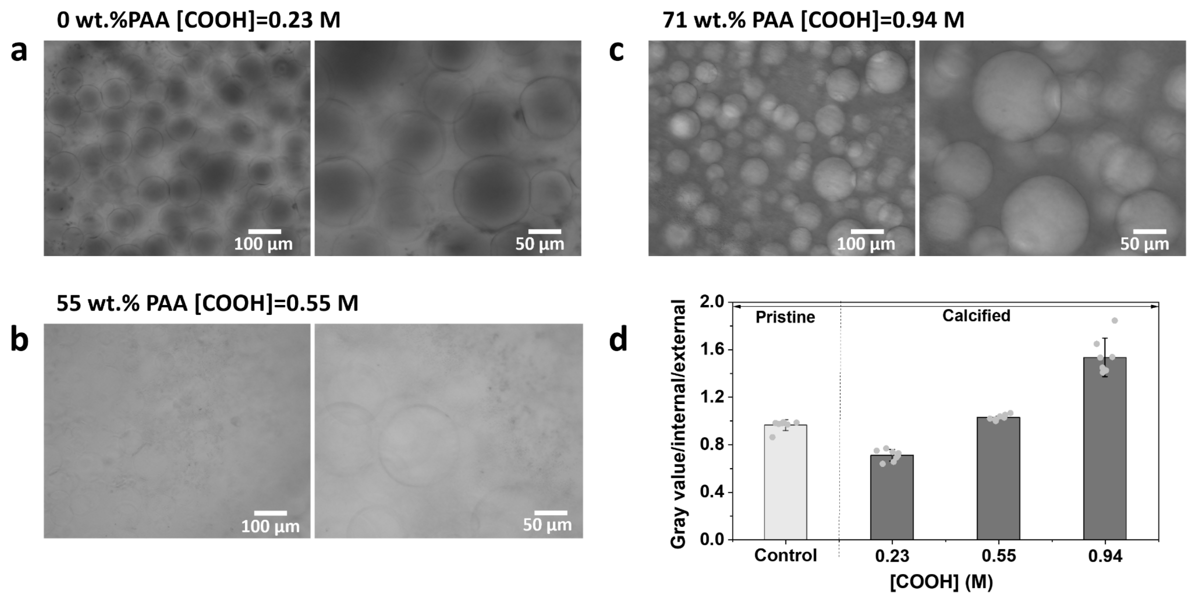

Supplementary Fig. 25. (**a-c**) Bright field (BF) images of calcified ALP-containing MA-colloidosome/Alg-MA/PAA prototissue films prepared with PAA proportions of 0 (**a**), 55 (**b**) and 71 wt.% (**c**) in the extra-protocellular matrix (corresponding total [COOH] = 0.23 M (Alg only), 0.55 M (Alg + 55 wt.% PAA) and 0.94 M (Alg + 71 wt.% PAA). In each case, films were mineralized for 24 h in the presence of CaGP. Calcification occurs both within the colloidosomes and matrix but with higher relative levels of colloidosome calcification (decreased light transmittance) in the absence of PAA (**a**). Mineralization occurs at a similar level in both colloidosomes and surrounding matrix (similar transmittance values) at PAA = 55 wt.% (**b**); preferential calcification of the matrix occurs at PAA = 71 wt.% (**c**). (**d**) Colloidosome: matrix gray value ratios for calcified prototissues constructed with different amounts of PAA in the Alg-MA matrix. Gray values derived from BF images were determined using ImageJ software. Lower gray values correspond to decreased light transmittance and increased calcification. No intra-protocellular PEG was used in the experiments. Data are presented as mean values ± s.d. (n = 3 samples). Source data are provided as a Source Data file.

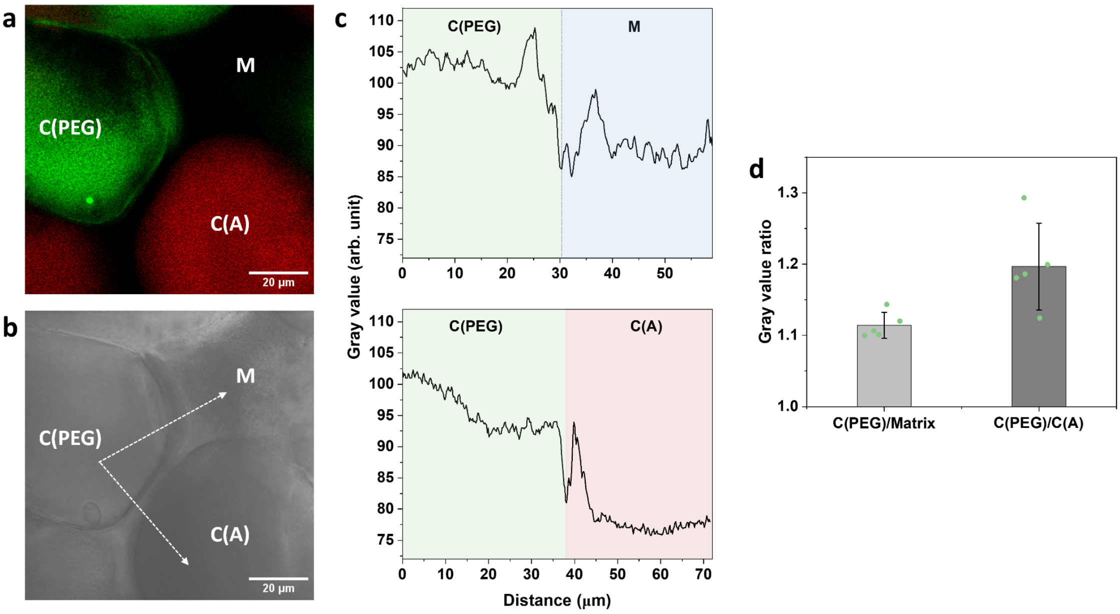

Supplementary Fig. 26. PEG-induced inhibition of intra-protocellular calcification in mineralized prototissues. (**a**) CLSM and (**b**) bright field (BF) images of a calcified prototissue comprising a binary population of MA-colloidosomes containing ALP/PEG (C(PEG) doped with FITC-labelled BSA (green fluorescence) or ALP (C(A) doped with Dy650 labelled-BSA (red fluorescence) and immobilized within a crosslinked Alg-MA matrix (M). (**c**) Line profiles (dashed lines in (**a**)) recorded from C(PEG) to M (top) or C(PEG) to C(A) (bottom). Decreases in gray value are associated with increased levels of mineralization. (**d**) Plot of mean gray value ratios showing different levels of site-specific calcification in the sequence C(PEG) < M < C(A). ACP formation is inhibited in the presence of PEG. Data are presented as mean values ± s.d. (n = 3 samples). Source data are provided as a Source Data file.

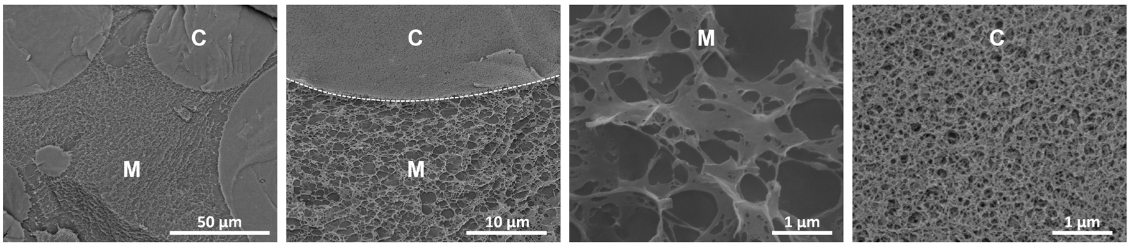

Supplementary Fig. 27. Cryo-SEM images of a non-calcified prototissue disk fabricated using ALP/PEG-encapsulated colloidosomes (C) and a Alg-MA/PAA matrix (M, Alg-MA : PAA = 30 : 70 weight ratio; $[PEG]_{IN}$ = 40 mg/mL). Networks of silica particles or a macroporous polymer scaffold are observed prior to calcification (phosphate production) in the C and M domains, respectively. Source data are provided as a Source Data file.

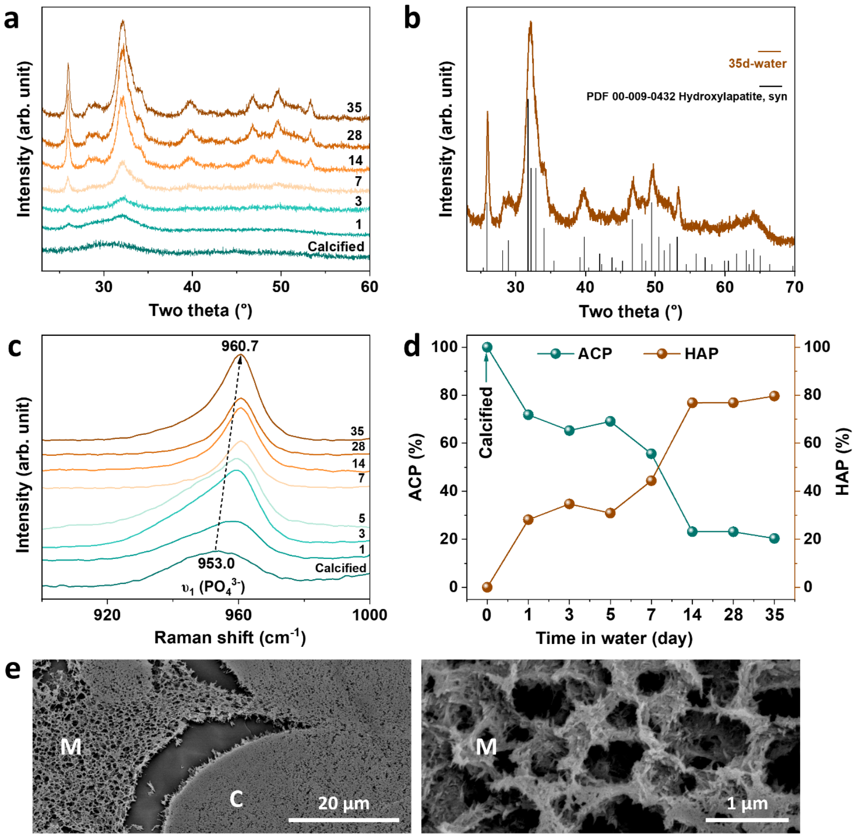

Supplementary Fig. 28. Time-dependent phase transformation of calcium phosphate in a selective extra-protocellular matrix mineralized prototissue. (**a**) Time-series of PXRD patterns of an extra-protocellular calcified prototissue disk stored in water over 35 d. The initial ACP phase starts to crystallize into poorly crystalline HAP within 1 d. (**b**) PXRD pattern of a calcified prototissue stored in water for 35 d compared to a standard diffraction pattern of HAP (synthesized, PDF 00-009-0432). (**c**) Time-series of Raman spectra of an extra-protocellular calcified prototissue disk stored in pure water for 35 d showing transformation of ACP to crystalline HAP over 35 d. The ʋ1 PO_4^{3-} band shifts from 953.0 cm^{-1} (ACP) to 960.7 cm^{-1} (HAP). Each spectrum is averaged from three tests on each sample. (**d**) Plot of the changes in percentage of ACP and HAP contents in an extra-protocellular calcified prototissue derived from Raman spectra in **c** by deconvolution analysis. (**e**) Cryo-SEM images of a freeze-fractured 24 h calcified prototissue after immersed in water for 35 d showing small needle-like HAP nanocrystals. Source data are provided as a Source Data file.

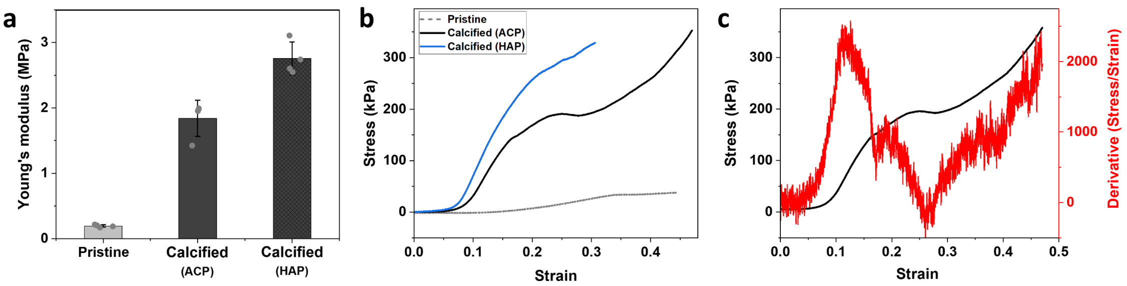

Supplementary Fig. 29. (**a**) Mechanical properties of non-mineralized (pristine, E = 0.2 MPa) and selective matrix-calcified MA-colloidosome/Alg-MA/PAA-integrated prototissues (24 h, calcified-ACP, E = 1.84 MPa; 35 d, calcified-HAP, E = 2.76 MPa) measured by unconfined universal compression. Data are presented as mean values ± s.d. (n = 4 samples). (**b**) Unconfined compression stress-strain curves of pristine (gray dashed line), calcified (ACP) (black line) and calcified (HAP) (blue line) MA-colloidosome/Alg-MA/PAA-integrated prototissues. (**c**) Overlapping plotting of stress-strain curve of calcified (ACP) MA-colloidosome/Alg-MA/PAA-integrated prototissue (black line) and corresponding derivative (red). Source data are provided as a Source Data file.

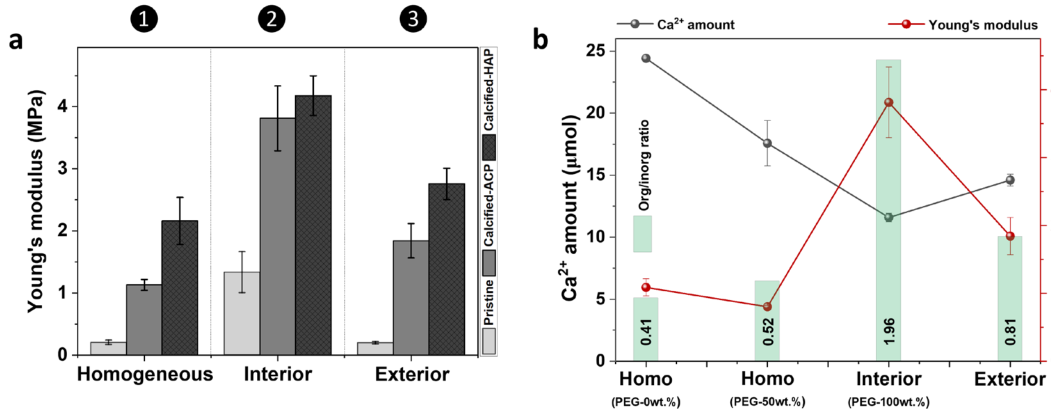

Supplementary Fig. 30. (**a**) Summary of Young's modulus (E) values of pristine prototissues (light gray) and calcified counterparts with different microstructures containing ACP (gray) or HAP (dark gray). Microstructures: ❶ homogeneous pristine and calcified prototissue, ❷ intra-protocellular calcified prototissues, and ❸ extra-protocellular matrix-calcified prototissues. (**b**) Plot of Ca^{2+} content and Young's moduli for different organic to inorganic ratios associated with different calcification regimes. Increased stiffness of the calcified prototissue is associated with a synergistic effect between the organic matrix and inorganic mineral. Source data are provided as a Source Data file.

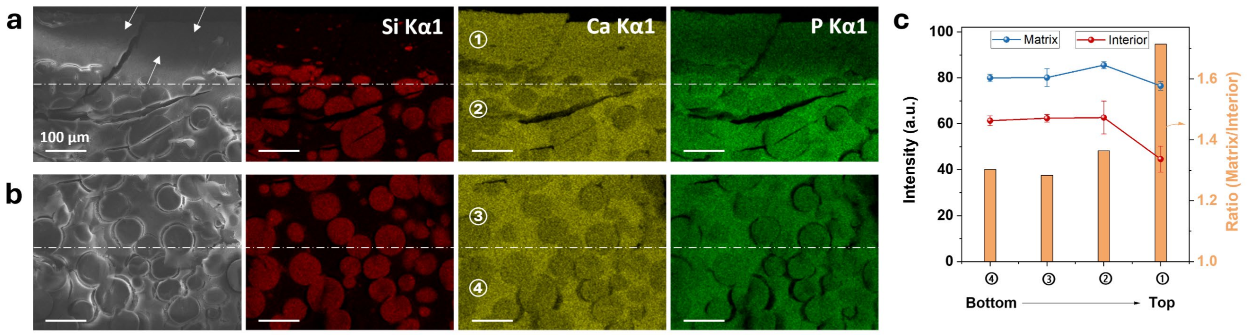

Supplementary Fig. 31. SEM-EDX element mapping analysis of a 24 h-calcified gradient prototissue fabricated after sedimentation for 5 min. (**a**) Top and (**b**) bottom zones showing secondary electron images (left) with corresponding element mapping for Si, Ca and P. (**c**) Plot of Ca intensity in both matrix and colloidosome interior in the four sub-zones ①②③④ and the corresponding Ca intensity ratio (matrix/interior). The slight decrease in Ca intensity in the matrix on the top layer ① is ascribed to the backwards position towards the EDX detector (a shadow marked by white arrow in **a**), which limits the Ca element detection. The results demonstrate that the average Ca content (Ca intensity in the matrix, colloidosome interior and colloidosome population density) increases from the bottom to top layer, confirming gradient calcification in the prototissue. Data are presented as mean values ± s.d. (n = 5 selected areas). Decreasing levels of Ca and P are detected in the top area of the sample, which could be due to the diffusion limitation of Ca and P into the top layer. The Ca/P ratio (2.48) in the top area is higher than the ratio measured in the calcified bottom region (2.02), which is attributed to the elevated Ca^{2+} levels associated with the matrix. Source data are provided as a Source Data file.

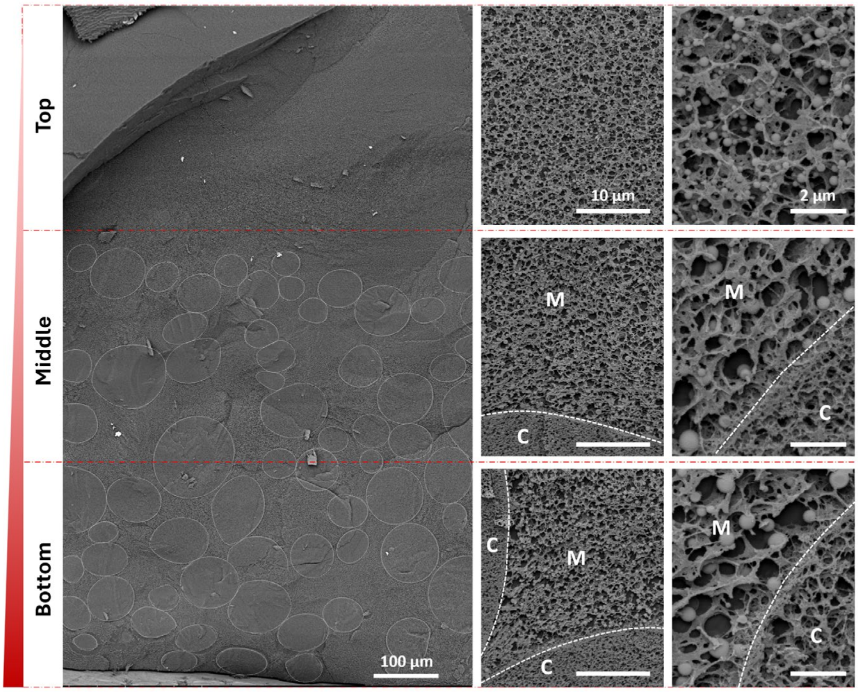

Supplementary Fig. 32. Cryo-SEM images of a calcified segregated prototissue fabricated after sedimentation for 10 min. Images from left, middle and right columns correspond to low, medium and high magnification images, respectively. Scale bars are 100, 10 and 2 μm, respectively. Images from top, middle and bottom rows correspond to the top, middle and bottom zones of the calcified prototissue, respectively. The top layer comprises a matrix-enriched layer without embedded colloidosomes while a high number density of colloidosomes is tightly packed at the bottom zone. The matrix of the segregated prototissue is highly calcified with spherical calcium phosphate nanoparticles while the colloidosome interior remains porous and non-calcified. The white dashed line indicates the membrane of colloidosome. M, matrix; C, colloidosome. Source data are provided as a Source Data file.

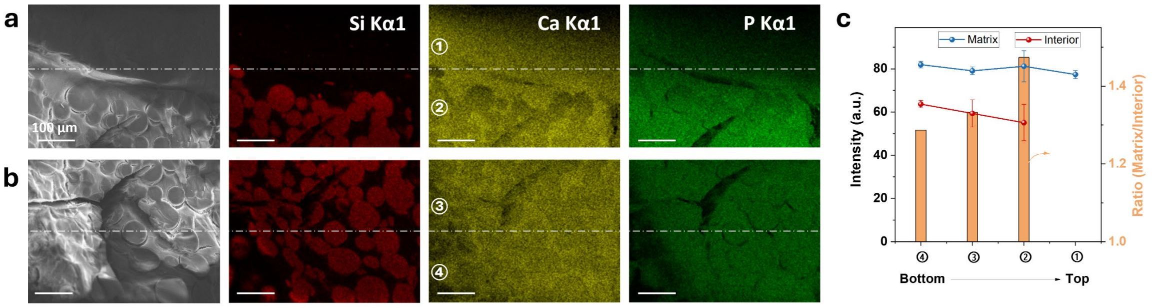

Supplementary Fig. 33. SEM-EDX element mapping analysis of a 24 h-calcified segregated prototissue fabricated after sedimentation for 10 min, (**a**) top and (**b**) bottom zones showing secondary electron images with corresponding element mapping for Si, Ca and P. (**c**) Plot of Ca intensity in both matrix and colloidosome interior in the four sub-zones ①②③④ and the corresponding Ca intensity ratio (matrix/interior). An increase in Ca intensity from the bottom to top is detected. Top ① zone is a calcified segregated matrix-enriched zone. The slight drop in Ca intensity in the matrix on the top layer ① is ascribed to backwards position towards the EDX detector (shadow in top ①), which limits Ca element detection. Data are presented as mean values ± s.d. (n = 5 selected areas). The calcified sample prepared after 10 min sedimentation showed similar characteristics to the calcified protottissue prepared after 5 min sedimentation (see Supplementary Fig. 31). The top area shows a higher Ca/P ratio (2.61), consistent with decreasing levels of P due to matrix-induced diffusion constraints into the top layer. Source data are provided as a Source Data file.

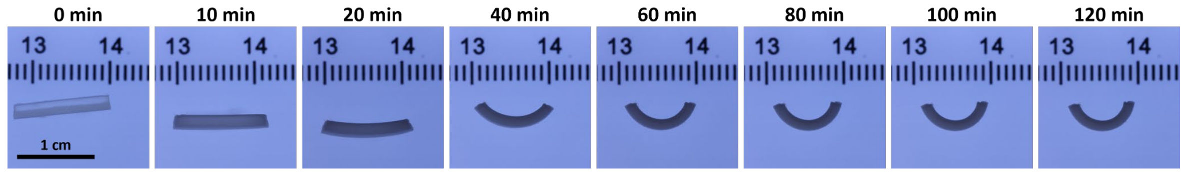

Supplementary Fig. 34. Time-series of photographs of a non-mineralized gradient prototissue strip after addition of 0.05 M $CaCl_2$ showing progressive bending over 120 min towards the matrix-enriched region. Initially (0 min) the prototissue is transparent and becomes increasingly opaque with Ca^{2+}-induced crosslinking. Large graduation marks, 1 cm. Source data are provided as a Source Data file.

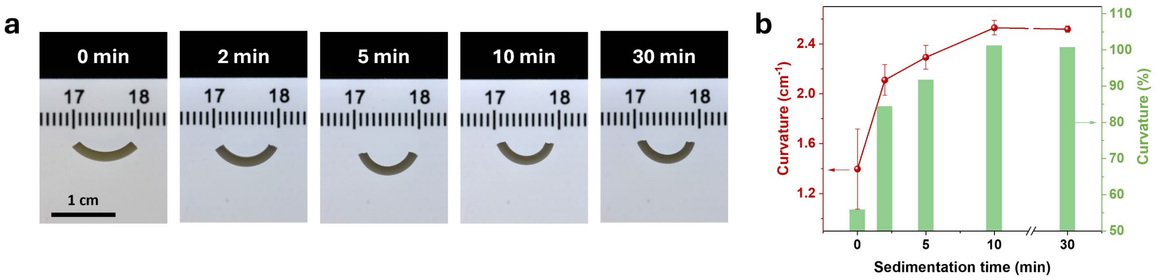

Supplementary Fig. 35. (**a**) Photographs of a series of 24 h-calcified gradient prototissues constructed by sedimentation for 0, 2, 5, 10 or 30 min. Large graduation marks, 1 cm. (**b**) Plot of curvatures of calcified prototissues as function of sedimentation time showing dependence of the chemo-mechanical response on the presence of a gradient/segregated calcified microstructure. Data are presented as mean values ± s.d. (n = 3 samples). Source data are provided as a Source Data file.

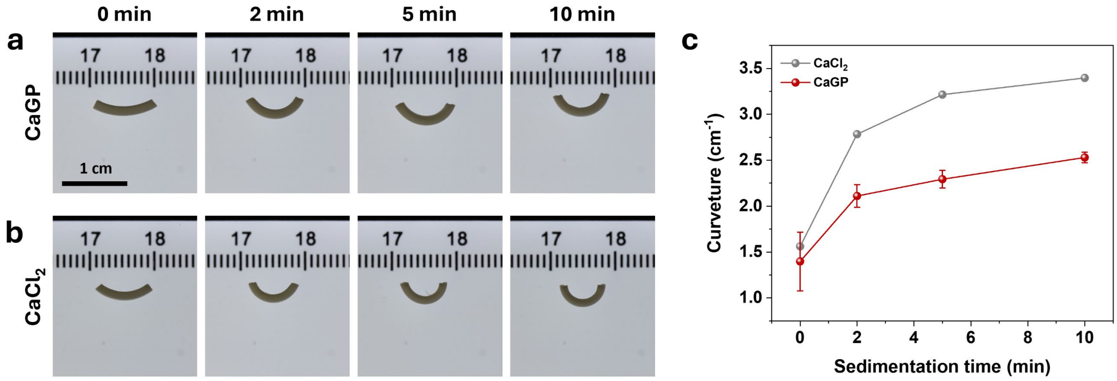

Supplementary Fig. 36. Photographs of (**a**) 24 h-calcified gradient prototissues and (**b**) Ca^{2+} ion-induced deformed prototissues constructed by sedimentation for 0, 2, 5 or 10 min. Large graduation marks, 1 cm. (**c**) Plots of curvature of calcified and Ca^{2+} ion-induced deformed prototissues as a function of sedimentation time. Data are presented as mean values ± s.d. (n = 3 samples). Source data are provided as a Source Data file.

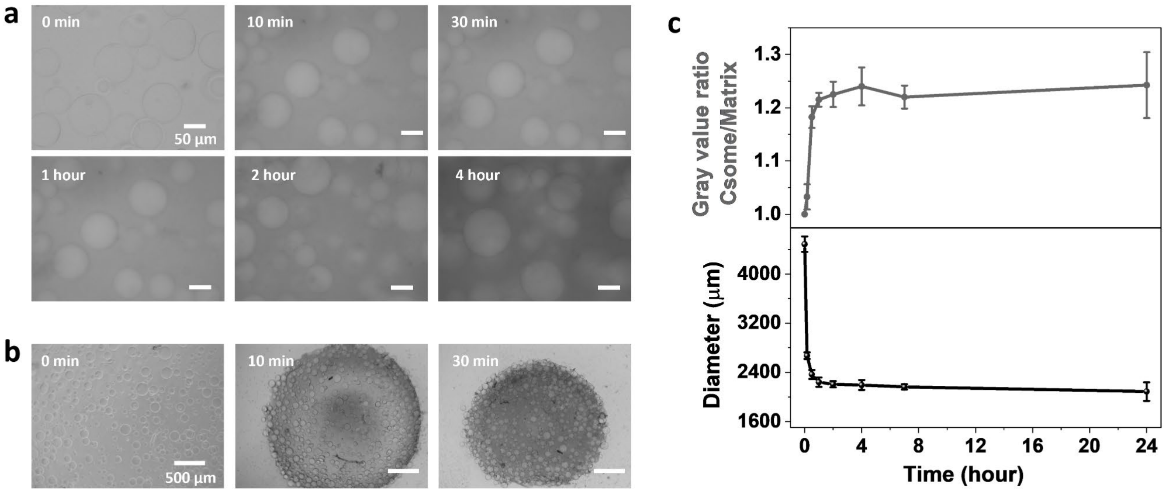

Supplementary Fig. 37. (**a**) High and (**b**) low magnification time-series of bright field microscopy images of a prototissue film prepared by photo-assisted integration of ALP-containing MA-colloidosomes within a covalently crosslinked Alg-MA/PAA (30 : 70 wt.% ratio), followed by immersion in 0.05 M $CaCl_2$ solution. Gradual darkening of the matrix is associated with progressive ionic crosslinking of the matrix; in contrast, the colloidosomes remain relatively transparent. Extensive ionic crosslinking of the matrix results in film shrinkage with a 54% decrease in the diameter. Scale bars in **a**, **b** are 50 µm and 500 µm respectively. (**c**) Plots of time-dependent gray value colloidosome (Csome)/matrix ratios (top) and change in diameter of the prototissue film (bottom). Extensive ionic crosslinking of the matrix results in an increase in the gray value Csome/matrix ratio from *ca.* 1 to 1.2 along with a 54% decrease in the diameter. Data are presented as mean values ± s.d. (n = 3 samples). Source data are provided as a Source Data file.

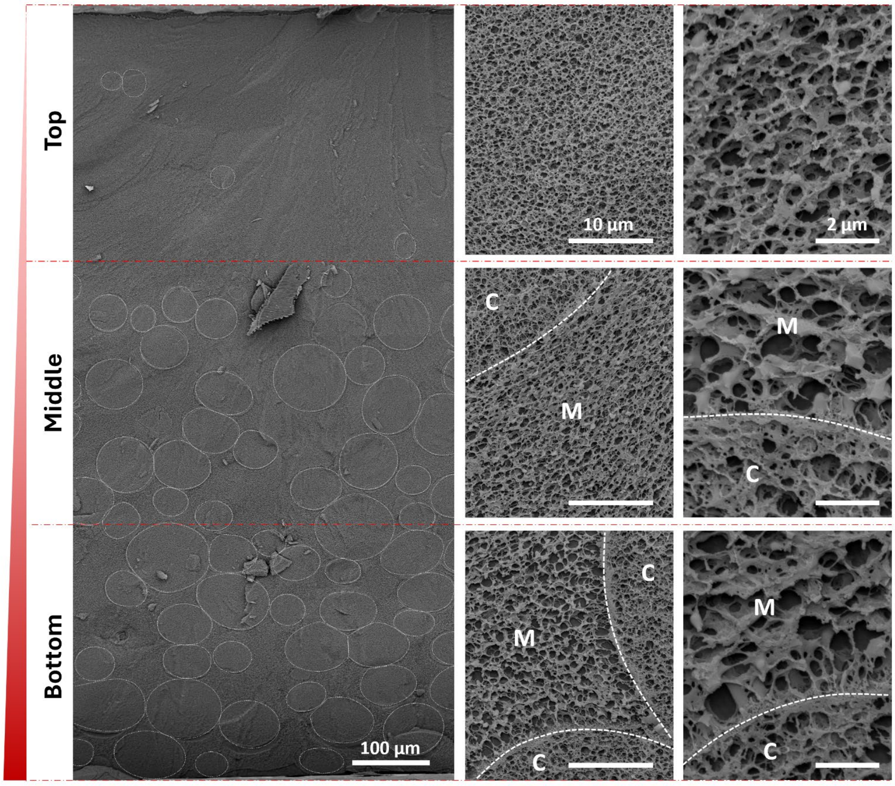

Supplementary Fig. 38. Cryo-SEM images of a $CaCl_2$ solution-immersed gradient prototissue fabricated after sedimentation for 5 min. Images from left, middle and right columns correspond to low, medium and high magnification images, respectively. Scale bars are 100, 10 and 2 µm, respectively. Images from top, middle and bottom rows correspond to the top, middle and bottom zones of the gradient prototissue respectively. Few colloidosomes are observed in the top zone. The colloidosome number density increases from the top to bottom zone with dense colloidosome packing in the bottom zone. No calcium phosphate mineralization was detected. The white dashed line indicates the colloidosome membrane. M, matrix; C, colloidosome. Source data are provided as a Source Data file.

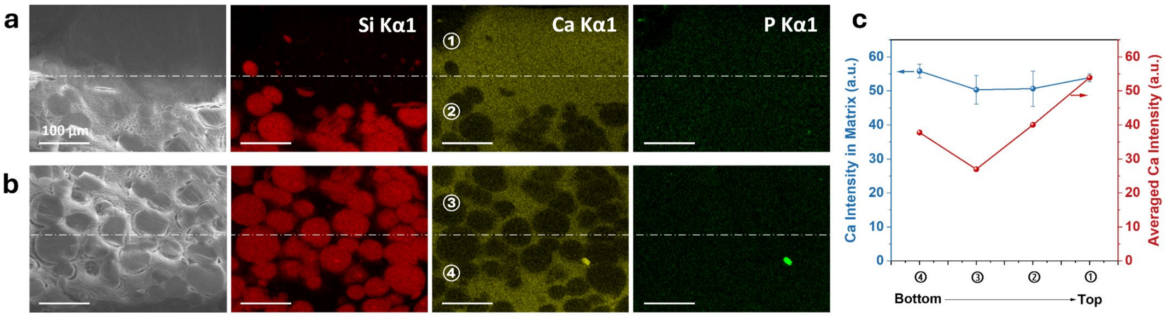

Supplementary Fig. 39. SEM-EDX element mapping analysis of a $CaCl_2$ solution-immersed gradient prototissue fabricated after sedimentation for 5 min. (**a**) Top and (**b**) bottom zones showing secondary electron images with corresponding element mapping for Si, Ca and P. Negligible levels of P are detected. (**c**) Plot of Ca intensity in the matrix in the four sub-zones ①②③④ and the corresponding averaged Ca intensity in each zone (Ca intensity in matrix multiplied by matrix volume fraction). The plots show the continuous increase of average Ca levels from the bottom to top zone in the composite. Negligible Ca is detected in the colloidosome interior. Data are presented as mean values ± s.d. (n = 5 selected areas). Minimal levels of P were detected, indicating the absence of calcium phosphate mineralization. The detected Ca was associated with the binding of Ca^{2+} ions to carboxyl groups in the Alg-MAA/PAA matrix. Source data are provided as a Source Data file.

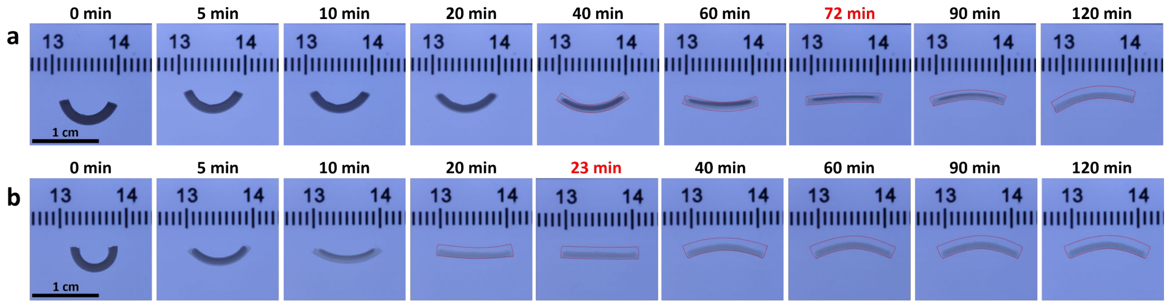

Supplementary Fig. 40. (**a**) Time-series of photographs of a deformed calcified gradient prototissue strip after immersion in 35 mL of 0.05 M EDTA at pH=7.4. The opaque bent strip gradually becomes straight at around 72 min when the colloidosome-enriched side becomes increasingly transparent while the matrix-enriched side partially remains opaque (i.e. calcified). With prolonged immersion time, the matrix side becomes translucent and exhibits reverse bending towards to the colloidosome-enriched side, which is attributed to swelling of the covalently crosslinked Alg-MA/PAA matrix. (**b**) As for (**a**) but for a deformed prototissue strip prepared by addition of 0.05 M $CaCl_2$. The opaque bent strip becomes straight within 23 min and then exhibits reverse bending towards to the colloidosome-enriched side. Dashed red lines identify the shape boundary. Large graduation marks, 1 cm. Source data are provided as a Source Data file.

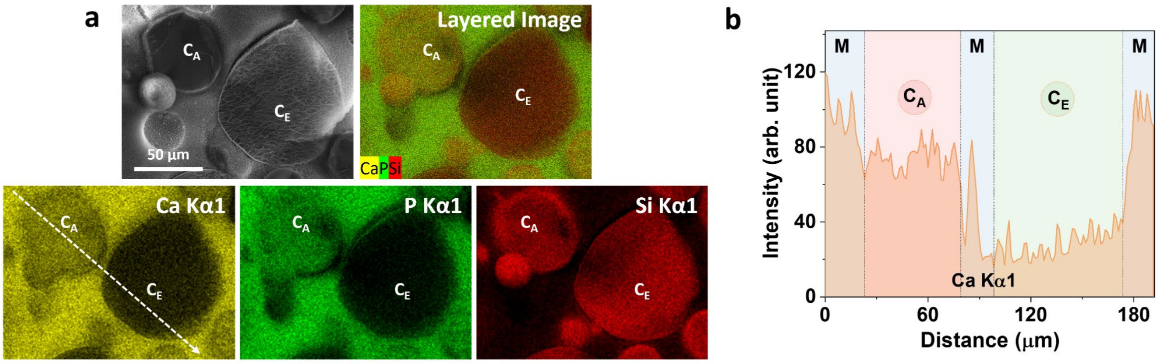

Supplementary Fig. 41. (**a**) SEM-EDX elemental mapping for a calcified multi-protocellular prototissue constructed from ALP-containing colloidosome (C_A) and esterase-containing colloidosomes (C_E) showing Ca/P/Si distributions. The highest Ca and P signals are detected in the Alg-MA matrix followed by in C_A and C_E. (**b**) Corresponding line profile of Ca distribution along the white dash line shown in **a**. Ca is present in both the matrix and C_A population, with the former being highly calcified. Lower levels of Ca are observed in the C_E population. Source data are provided as a Source Data file.

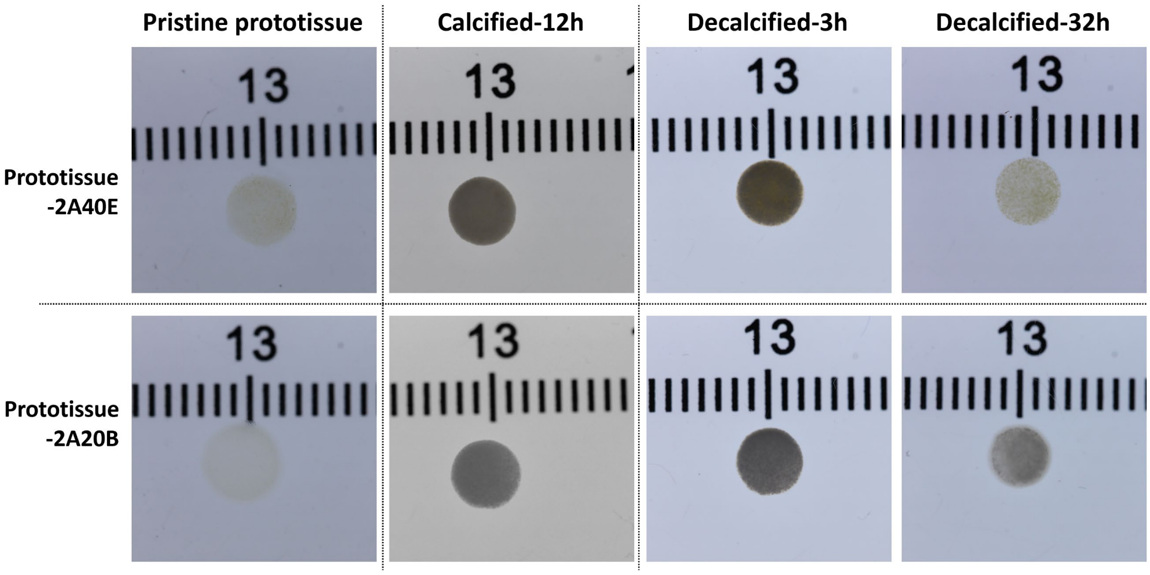

Supplementary Fig. 42. Photos showing protocell-mediated calcification-decalcification sequence in prototissue disks consisting of a Alg-MA matrix-integrated 1 : 1 binary population of MA-colloidosomes containing either ALP or esterase (top row; 2 mg/mL ALP; 40 mg/mL esterase; prototissue-2A40E), or either ALP or BSA (bottom row; 2 mg/mL ALP, 20 mg/mL BSA; prototissue-2A20B). In both cases, the translucent pristine prototissues become opaque after 12 h after addition of CaGP and endogenous ALP-mediated calcification. Transfer of the calcified multi-protocellular prototissue disks into 1 mL of 0.5 M ethyl acetate in 5 mM Tris buffer (pH, 7.5) gives rise to progressive decalcification over 32 h for prototissue-2A40E. The decalcified disk remains intact and becomes translucent. In contrast, calcified prototissue-2A20B remains opaque after 12 h. Additional results showed that the BSA concentration had no effect on the decalcification results. Source data are provided as a Source Data file.

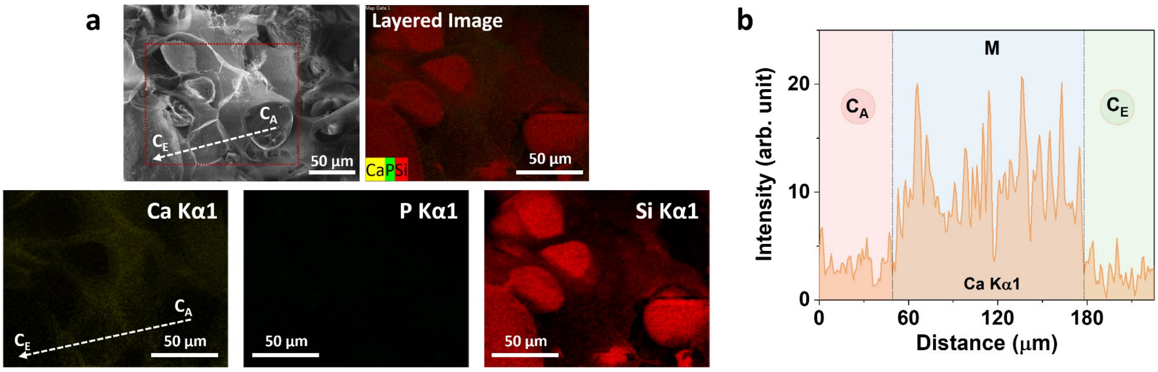

Supplementary Fig. 43. (**a**) SEM-EDX elemental mapping for a decalcified multi-protocellular prototissue constructed from ALP-containing colloidosome (C_A) and esterase-containing colloidosomes (C_E) showing Ca/P/Si distributions. Limited levels of Ca and P are detected in the Alg-MA matrix and within the two colloidosome populations. Si is detected in both C_A and C_E populations. (**b**) Corresponding line profile of Ca distribution recorded along the white dashed line shown in **a**. The marginally high Ca level in the matrix is associated with Ca crosslinking of the Alg-MA chains. Source data are provided as a Source Data file.

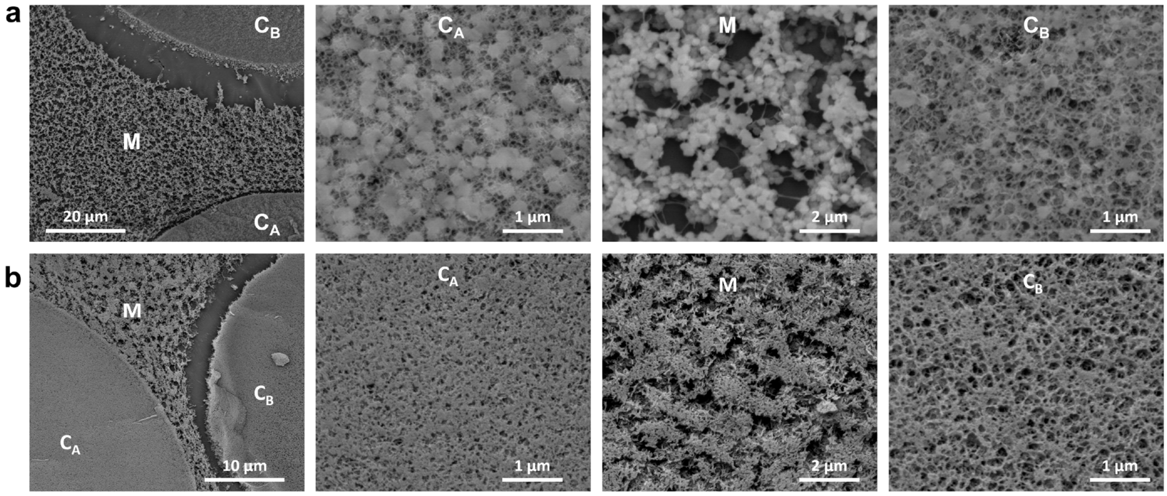

Supplementary Fig. 44. Cryo-SEM images of 12-h calcified prototissues prepared with MA-colloidosomes containing either 2 mg/mL ALP (C_A) or 20 mg/mL BSA (C_B) before (**a**) and 32 h after (**b**) addition of ethyl acetate. From left to right; images recorded across C_A/M/C_B interfaces (M, Alg-MA matrix), within C_A, M or C_B. High-density networks of ACP and silica particles are observed in calcified C_A and C_B with the former being more heavily mineralized. Calcification in M produces a high-density network of ACP particles. These structures remain in place in (**b**) except that the ACP particles transform into short rod-shaped particles due to exposure to water for 32 h. Source data are provided as a Source Data file.

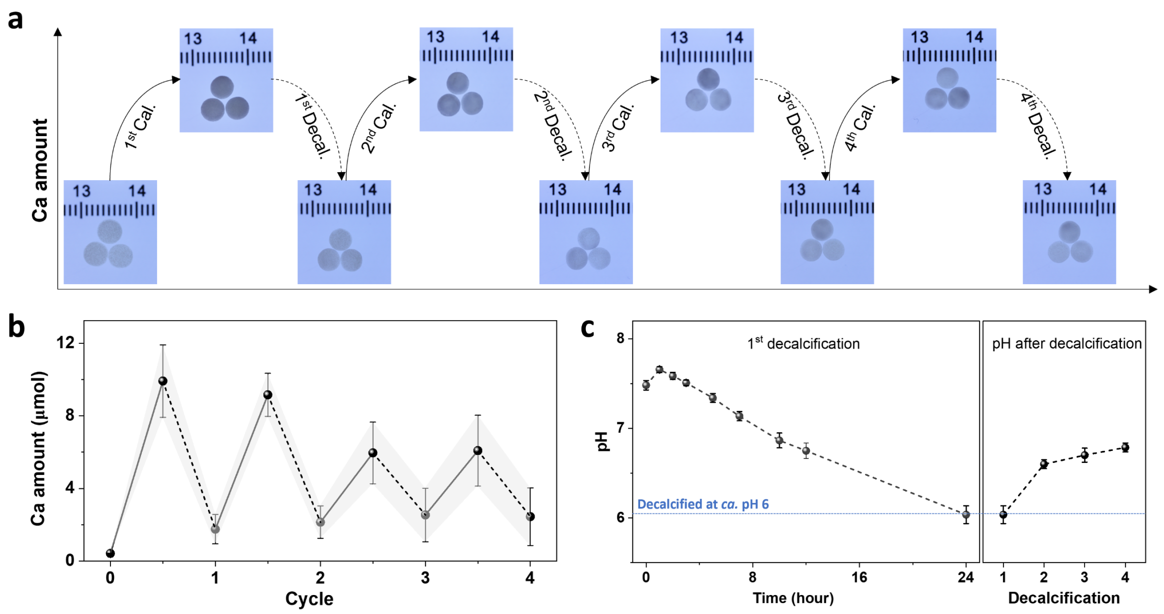

Supplementary Fig. 45. Cycling of calcification/decalcification in multi-protocellular prototissues. (**a**) Time series of photographs of calcified/decalcified (Cal./Decal.) prototissues recorded at the end of each calcification/decalcification step showing the gradually decrease in the degree of calcification (top series of photos, reduced darkness) and increase in residual calcium phosphate mineral (bottom series of photos, increased darkness). (**b**) Plot of calcium content in the pristine prototissue (cycle 0) and in the prototissue at the end of each calcification/decalcification step showing the progressive decrease in total calcium content and increase in residual Ca at the end calcification/decalcification cycles 2, 3 and 4. The mineral precipitated in the prototissue during the 3rd and 4th cycles is 60 wt.% of the value determined for cycle 1. Data are presented as mean values ± s.d. (n = 3 samples). (**c**) *Left*: Time-dependent plots of pH during the 1st decalcification step showing a decrease in pH to *ca.* 6 after 24 h. *Right*: plot of pH at the end of each decalcified step (1-4) showing a gradual increase of pH, indicating a gradual loss in the capacity of the embedded colloidosomes to generate protons. Data are presented as mean values ± s.d. (n = 3 samples). Source data are provided as a Source Data file.

3. Supplementary Tables

Supplementary Table 1. Thermogravimetric analysis: weight percentage of components in uncalcified prototissues (ref.) and calcified prototissues. "C*X*A-*Y*": C, colloidosome; A: ALP; *X*: ALP concentration in mg/mL; *Y*: calcification time period. For example, "C2AP40" refers to colloidosomes containing 2 mg/mL ALP enzyme and 40 mg/mL PEG. Data are presented as mean values ± s.d. (n = 3 samples).

Prototissue	Water (wt.%)	Organic (wt.%)	Inorganic (wt.%)	Silica (wt.%)	CaP (wt.%)	Organic /inorganic
**Scenario 1: Homogeneous calcified prototissue (Csome/Alg-MA integrated prototissue)**						
**C2A-PEG0-ref.**	5.98 ± 1.41	36.99 ± 0.25	57.19 ± 1.63		-	
**C2A-PEG0-12h**	8.62 ± 0.15	27.36 ± 0.49	64.01 ± 0.64	42.41 ± 0.76	21.61 ± 1.40	
**C2A-PEG0-1d**	9.05 ± 0.20	26.24 ± 1.01	64.71 ± 0.84	40.67 ± 1.57	24.05 ± 2.41	0.41
**C2A-PEG0-2d**	9.96 ± 0.43	25.33 ± 1.10	64.71 ± 0.84	39.26 ± 1.72	25.45 ± 2.53	
**C2A-PEG0-3d**	11.60 ± 2.21	26.38 ± 0.37	62.03 ± 1.84	40.88 ± 0.57	21.14 ± 1.26	
**C5A-PEG0-1d**	10.31 ± 0.16	16.24 ± 0.32	73.44 ± 0.48	25.17 ± 0.49	47.95 ± 0.98	
**C10A-PEG0-1d**	11.13 ± 0.26	11.28 ± 0.26	77.59 ± 0.06	17.48 ± 0.41	60.11 ± 0.42	
**C20A-PEG0-1d**	12.46 ± 1.64	12.23 ± 0.10	75.30 ± 1.75	18.96 ± 0.16	56.34 ± 1.91	
**C30A-PEG0-1d**	10.96 ± 0.45	13.16 ± 0.52	75.88 ± 0.97	20.40 ± 0.80	55.48 ± 1.77	
**C2A-PEG50-ref**	3.68 ± 1.44	52.99 ± 0.02	43.33 ± 1.43		-	
**C2A-PEG50-1d**	8.88 ± 0.36	31.34 ± 0.48	59.78 ± 0.11	25.69 ± 0.39	34.09 ± 0.50	0.52
**Scenario 2: Intra-protocellular calcified prototissue (Csome/PEGDM integrated prototissue)**						
**C2A-ref.**	0.78 ± 0.80	85.20 ± 0.32	14.02 ± 1.12		-	
**C2A-1d**	9.56 ± 1.52	59.84 ± 2.03	30.59 ± 0.51	9.84 ± 0.33	20.75 ± 0.84	1.96
**Scenario 3: Matrix calcified prototissue (Csome/Alg-MA/PAA integrated prototissue)**						
**C2AP40-ref.**	2.50 ± 0.08	55.03 ± 0.41	42.47 + 0.49		-	
**C2AP40-1d**	8.36 ± 0.10	40.91± 1.40	50.73 ± 1.50	31.47 ± 1.07	19.27 ± 2.57	0.81

Density of calcium phosphate, 2.01 ± 0.13 g cm^{-3}.

Supplementary Table 2: SEM-EDX analysis of different calcified prototissues.

	Ca	P	O	C	Si	Ca/P
**Supplementary Fig. 8, Alg-MA/Csome**	7.0	3.8	14.1	7.6	1.6	1.84
**Fig. 3, PEGDM/Csome; PEG 100wt%**	9.9	6.2	29.6	52.2	0.5	1.60
**Fig. 4, Alg-MA/PAA/Csome**	17.4	8.6	39	22.3	6.2	2.02
**Supplementary Fig. 31, Gradient 5 min, Top**	7.2	2.9	16.3	14.4	1.4	2.48
**Supplementary Fig. 31, Gradient 5 min, Bottom**	8.0	4.0	15.0	11.7	2.1	2.00
**Supplementary Fig. 33, Gradient 10 min, Top**	6.0	2.3	23	18.7	1.1	2.61
**Supplementary Fig. 33, Gradient 10 min, Bottom**	6.2	3.2	18.9	13.3	3.2	1.94
**Supplementary Fig. 39, Gradient 5 min ref, Top**	3.8	0.0	18.8	20.0	1.9	-
**Supplementary Fig. 39, Gradient 5 min ref, Bottom**	2.9	0.0	19.7	21	5.3	-
