# Supplementary material for: Cytomimetic calcification in chemically self-regulated prototissues
Source: Nat Commun. 2025 May 3;16:4138. doi: 10.1038/s41467-025-59251-x (PMC12049547; doi:10.1038/s41467-025-59251-x)
Supplement: Supplementary file 2 — Description of Additional Supplementary Files [file 41467_2025_59251_MOESM2_ESM.pdf]

Description of Additional Supplementary Files

File name: Supplementary Movie 1

Description: Gradient calcification process associated with chemomechanical transduction within a prototissue (x300 speed)

A prototissue with a colloidosome number density gradient was exposed to 20 mL of 0.05 M CaGP solution (0.2 M Tris buffer pH=7.5). Calcification associated with chemomechanical transduction within the prototissue was recorded at a time interval of 1 min for 120 min.

File name: Supplementary Movie 2

Description: EDTA-mediated dissolution of calcium phosphate in a calcified prototissue (x323 speed)

A calcified gradient prototissue (calcified for 24 hours) was immersed in 35 mL 0.05 M EDTA aqueous solution (pH=7.4). Gradual dissolution of the inorganic mineral in the calcified prototissue in the presence of EDTA was recorded at a time interval 1 min for up to 180 min.
